# Massive release of predominantly magmatic carbon drove an Early Jurassic hyperthermal

**DOI:** 10.1038/s41467-026-77747-y

**Published:** 2026-09-18

**Authors:** Wenhan Chen, Yuyang Wu, David B. Kemp, Ying Cui, Alexandre Pohl, Wolfgang Ruebsam, Dongyu Zheng, Chao Li

**Affiliations:** 1 State Key Laboratory of Oil and Gas Reservoir Geology and Exploitation & Institute of Sedimentary Geology, Chengdu University of Technology, Chengdu, China; 2 International Center for Sedimentary Geochemistry and Biogeochemistry Research, Chengdu University of Technology, Chengdu, China; 3 College of Marine Science and Technology, China University of Geosciences, Wuhan, China; 4 State Key Laboratory of Geomicrobiology and Environmental Changes and Hubei Key Laboratory of Critical Zone Evolution, School of Earth and Planetary Sciences, China University of Geosciences, Wuhan, China; 5 Department of Earth and Environmental Studies, Montclair State University, Montclair, NJ USA; 6 Université Bourgogne Europe, CNRS, Biogéosciences UMR 6282, Dijon, France; 7 Department of Organic and Isotope Geochemistry, Institute of Geoscience, Kiel University, Kiel, Germany

**Keywords:** Palaeoceanography, Palaeoclimate, Marine chemistry

## Abstract

The Toarcian Oceanic Anoxic Event (T-OAE, ~183 Ma) was a major hyperthermal event in the Phanerozoic, characterised by massive carbon release and widespread organic carbon burial. However, the source(s), flux, and sequestration pathways of carbon remain uncertain. Here, we assimilate globally representative carbon isotope and atmospheric CO_2_ records into an Earth system model to decipher the carbon cycle during the T-OAE. Our simulations indicate the release of ~18,000 Pg C of predominantly magmatic carbon through the T-OAE, though with an input of carbon from thermogenic and/or biogenic sources at the onset of the event. We show that at least 6800 Pg of organic carbon was buried during the event, which, combined with silicate weathering-driven excess carbon consumption (~6000 Pg C), helped the Earth’s climate system to recover from large-scale carbon release. Our results provide quantitative constraints on the tempo, magnitude, and recovery mechanisms of carbon-cycle perturbations during extreme greenhouse intervals.

## Abstract (web summary)

Earth system modelling indicates that around 18,000 Pg C of predominantly magmatic carbon drove the Early Jurassic Toarcian hyperthermal event, while organic carbon burial and silicate weathering helped restore the climate system.

## Introduction

The Toarcian Oceanic Anoxic Event (T-OAE, ~183 Ma) was a brief interval of pronounced global warming in the Early Jurassic^1^. The event is typically marked in the stratigraphic record by an abrupt negative carbon isotope (δ^13^C) excursion (N-CIE) in both organic and inorganic carbon substrates^2,3^. This excursion is interpreted as a diagnostic signal of massive ^13^C-depleted carbon release into the ocean–atmosphere system^2^. This carbon release triggered global warming and a cascade of environmental responses, including widespread accumulation of organic carbon (OC)-rich deposits, oceanic deoxygenation, and severe perturbations to hydrological cycling and continental weathering^1,4–6^.

The detailed patterns of carbon release and sequestration that respectively triggered and terminated the T-OAE are debated^2,7–13^. The emplacement of the Karoo–Ferrar Large Igneous Provinces (LIPs) in southern Gondwana has been proposed as a primary carbon source, either through volcanic degassing of CO_2_ during eruptions^7^ or via thermogenic carbon release associated with magma injection into OC-rich country rocks^8^. In addition, multiple δ^13^C records exhibit stepwise declines during the T-OAE N-CIE interval, implying pulsed carbon release^9–12^. The apparent regularity of these pulses has been interpreted as an astronomical control on carbon release, suggesting that at least some of the carbon released during the T-OAE was sourced from climatically sensitive surficial reservoirs (e.g., biogenic carbon stored in continental shelf methane hydrates, wetlands, or permafrost soils)^9–12^. Uncertainties in carbon sources hinder robust quantifications of carbon-release masses and rates, an issue further exacerbated by poor constraints on atmospheric CO_2_ levels (*p*CO_2_) and the timescale and magnitude of the T-OAE N-CIE (Supplementary Text 1).

Enhanced OC burial during the T-OAE, inferred from widespread accumulation of black shales, is commonly invoked as a key mechanism driving the carbon-cycle recovery^4^. However, proxy-based quantitative estimates of global OC burial during the T-OAE remain highly uncertain (ref. ^13^; see also Supplementary Text 1). Additionally, Os-isotope records suggest that rates of continental silicate weathering may have increased by 200–500% through the event^5,14,15^, consuming excess CO_2_ and likely contributing to climate recovery. Nevertheless, quantifying the magnitude of CO_2_ drawdown associated with enhanced continental silicate weathering remains challenging due to large uncertainties in weathering flux estimates^5^.

**Fig. 1:**
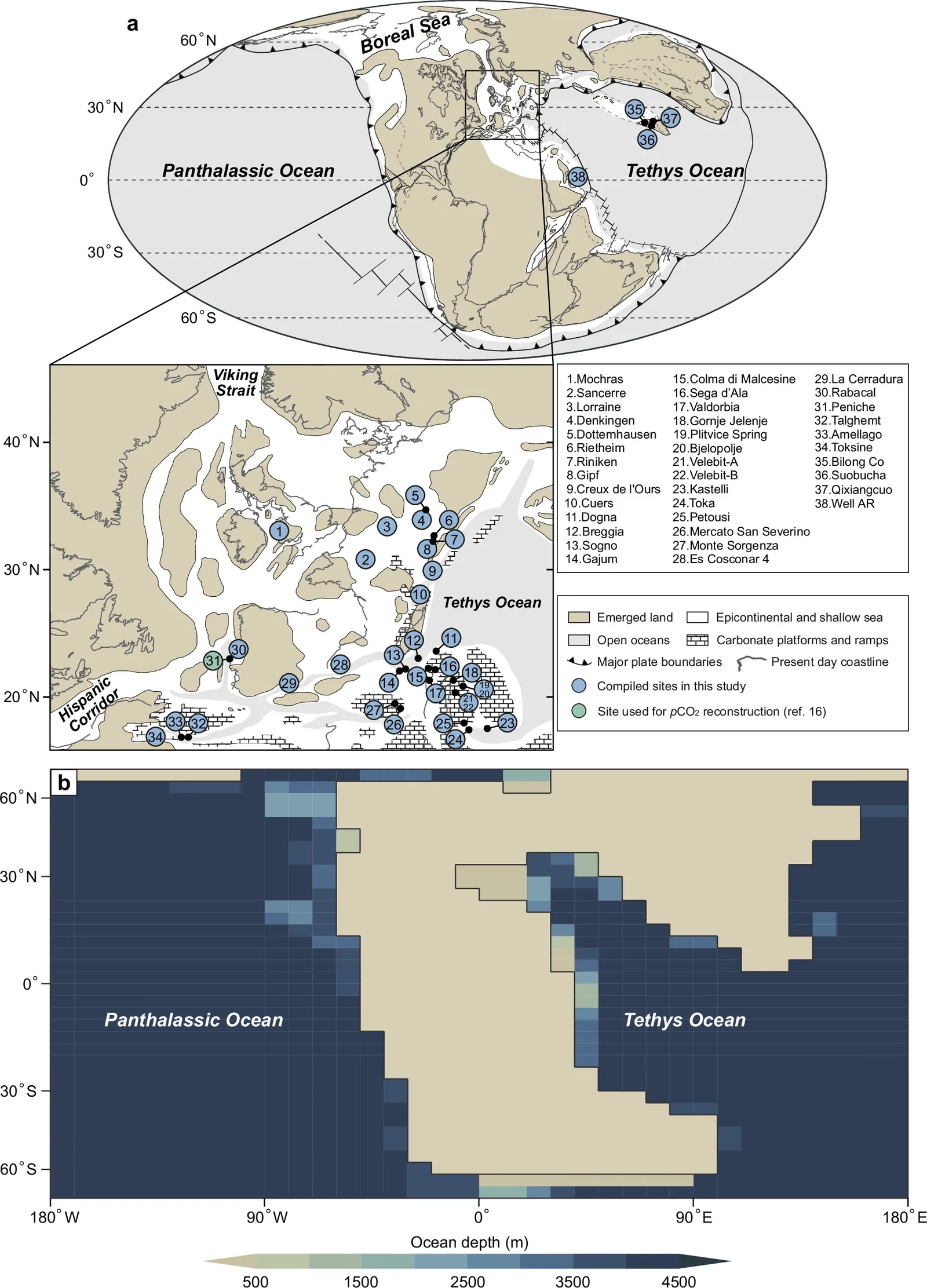
Geological setting during the Early Jurassic. **a** Palaeogeographical map of the Early Jurassic Toarcian Stage (~183 Ma, modified from ref. ^13^, reproduced with permission from Elsevier) showing the location of the studied sites used to build our composite δ^13^C_atm_ profile (references for the original δ^13^C_carb_ records used are provided in Supplementary Table 1). The inset map shows the palaeogeography of Europe and North Africa (area in black box on the global map). The underlying palaeogeographic map material was adapted from ref. ^90^ under a Creative Commons Attribution 4.0 International License (CC BY 4.0). **b** Palaeogeography and palaeobathymetry of the Early Jurassic Toarcian Stage used in cGENIE (modified from ref. ^81^ under CC BY 4.0).

**Fig. 2:**
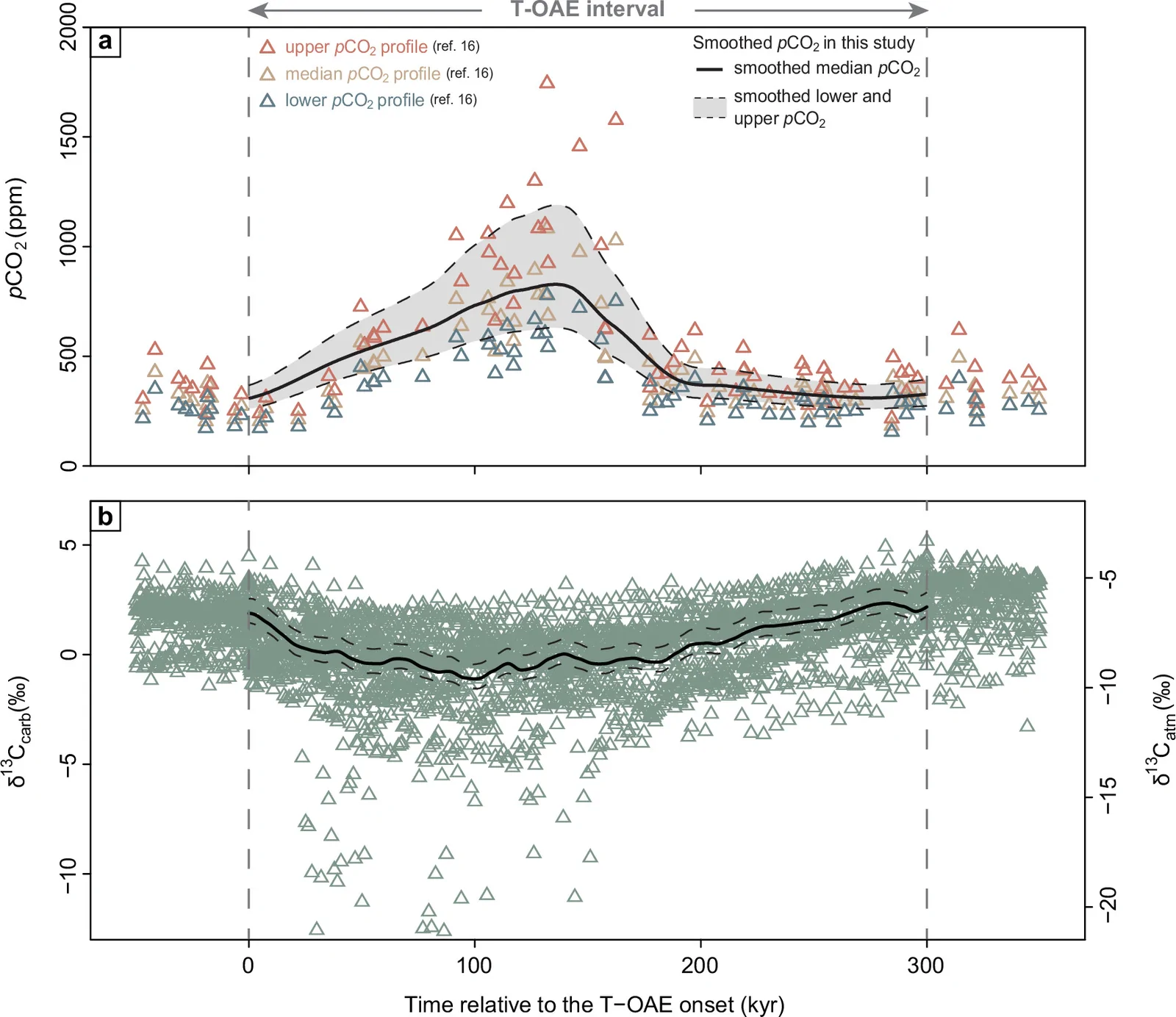
Compiled input data used for cGENIE simulations. **a** Reconstructed upper (84^th^ percentile), median, and lower (16^th^ percentile) *p*CO_2_ profiles across the T-OAE, based on the C_3_ plant CO_2_ proxy from Peniche, Portugal^16^, tied to a recently published high-precision geochronology for the T-OAE^17^. The *p*CO_2_ data used for the simulations are the smoothed profiles (solid and dashed lines) obtained by a LOESS fit method (see “Methods”). **b** δ^13^C_carb_ and converted δ^13^C_atm_ data (see main text) across the T-OAE, compiled from the 38 profiles shown in Fig. 1a and tied to the geochronology. The δ^13^C_atm_ data used for the simulations are smoothed (solid line) by a LOESS fit method (see “Methods”). The 95% confidence interval is constrained by the dashed lines. The two vertical grey dashed lines in both panels a and b define the interval of the T-OAE N-CIE (300 kyr)^17^. *T-OAE* Toarcian Oceanic Anoxic Event; *N-CIE* negative carbon isotope excursion.

Collectively, uncertainties in both carbon release and sequestration inhibit our ability to contextualise the T-OAE against anthropogenic emissions and climate warming, thus limiting its utility as a potentially important analogue for future climate change. To address this issue, we establish a globally representative profile of the T-OAE N-CIE by compiling carbonate carbon isotope records (δ^13^C_carb_) from 38 well-constrained marine sections (Fig. 1) and integrating them with a high-resolution *p*CO_2_ record^16^ and a refined geochronological framework^17^ (Fig. 2a, b). By assimilating these data into an Earth system model (cGENIE), we provide quantitative estimates of carbon release and removal during the T-OAE.

## Results and discussion

### High-resolution records of δ^13^C_carb_ and *p*CO_2_ during the T-OAE

The compiled high-resolution δ^13^C_carb_ records are from 38 bio- and chemo-stratigraphically constrained sites in the Tethys Ocean (Fig. 1a) where the T-OAE N-CIE has been unambiguously identified (Supplementary Fig. 1a; references in Supplementary Table 1). δ^13^C_carb_ values (*n* = 3159) during the T-OAE N-CIE interval range from −12.52‰ to +5.27‰ (median +0.45‰; Supplementary Fig. 2a), with ~95% of data falling within the range of −3‰ to +4‰ (Supplementary Fig. 2b). The N-CIE in each record is demarcated by an onset interval (marked by generally decreasing δ^13^C) and a recovery interval (generally increasing δ^13^C)^18^. The δ^13^C_carb_ profiles were stacked by aligning the N-CIE onset and termination in each record and defining the ages of these horizons as 0 kyr and 300 kyr, respectively, based on a T-OAE duration of ~300 kyr obtained recently using high-precision radioisotopic geochronology^17^ (see “Methods”).

Based on differences of the T-OAE N-CIE magnitude recorded in C_3_ land plants and marine substrates from the Peniche section (Portugal; site 31 in Fig. 1a), a relatively high-resolution atmospheric *p*CO_2_ record (*n* = 109) has recently been generated^16^, showing a marked *p*CO_2_ rise from ~280 + 50/ − 40 ppm to ~1100 + 700/ − 300 ppm through the event (Fig. 2a). This *p*CO_2_ profile was tied to our age model for compiled δ^13^C_carb_, aided by the fact that the Peniche record preserves an unambiguous expression of the N-CIE (see “Methods”). To account for uncertainties associated with regional changes in vegetation types and other environmental factors, Nordt et al.^16^ defined lower, median, and upper *p*CO_2_ profiles using the 16^th^, 50^th^, and 84^th^ percentiles of the estimated *p*CO_2_ values from Peniche (Fig. 2a), yielding a 68% confidence interval.

### Quantifying the T-OAE carbon cycle using Earth system modelling

Previous quantifications of the T-OAE carbon cycle have typically relied on box models within a forward-modelling framework^19–21^, in which an ensemble of predefined carbon injection scenarios is imposed, and the best-fit scenario is determined by comparing model outputs with geological observations (e.g., reconstructed *p*CO_2_, sea surface temperature (SST), and N-CIE morphology and magnitude). This approach requires a priori assumptions about carbon sources, typically treating them as invariant through time, and leads to non-unique solutions^19^. Moreover, poor constraints on *p*CO_2_ and the N-CIE morphology associated with carbon release through the T-OAE introduce substantial uncertainties in model–data comparison, leading to contrasting interpretations among studies. For example, Beerling and Brentnall^19^ considered biogenic methane as the main carbon source, while Ullmann et al.^20^ noted a significant role for a magmatism-derived carbon source. A recent Earth system modelling study using GEOCLIM suggested pulsed thermogenic carbon release as the major carbon source, without the need to invoke a biogenic carbon source such as methane hydrate^22^. By contrast, Kasemann et al.^21^ and Qiu et al.^12^ instead emphasised the necessity of isotopically lighter carbon (e.g., methane hydrate) as an additional carbon source to reproduce the observed N-CIE.

Here, we employ an improved modelling approach using the spatially resolved Earth system model of intermediate complexity cGENIE (see “Methods”). A double-inversion approach, consisting of forcing the atmosphere simultaneously with paired atmospheric carbon isotopes (δ^13^C_atm_) and *p*CO_2_, allows for a unique solution for the δ^13^C of the carbon source (δ^13^C_source_). It enables reconstructions of the masses and rates of carbon release and sequestration.

To prepare the necessary δ^13^C_atm_ input for cGENIE, we first convert our stacked δ^13^C_carb_ record (which reflects marine dissolved inorganic carbon) to a corresponding record of δ^13^C_atm_ by assuming an isotopic fractionation between the atmosphere and sedimentary carbonate of −8.5‰^23^. This approach is consistent with previous modelling work on carbon-cycle perturbations during other hyperthermal events, including the End-Permian mass extinction^24^ and OAE 1a in the Early Cretaceous^25^. A LOESS fit method was then used to smooth both the δ^13^C_atm_ profile and the *p*CO_2_ record to capture the overall trends and achieve synchronised time-step alignment (i.e., paired data) for the subsequent simulations (Fig. 2a, b; see “Methods”).

We adopt the smoothed median *p*CO_2_ profile (solid line in Fig. 2a) as the standard experiment (experiment 2; Supplementary Table 2). Additional simulations using the smoothed lower and upper *p*CO_2_ profiles (dashed lines in Fig. 2a; experiments 1 and 3, respectively) were conducted to quantify the influence of *p*CO_2_ uncertainty on the model outputs. Importantly, due to the potential attenuation of *p*CO_2_ changes arising from our smoothing of the input *p*CO_2_ record, we also performed an end-member sensitivity experiment (experiment 4) using an alternative *p*CO_2_ curve constrained by the maximum value (~1750 ppm) within the upper (84^th^ percentile) reconstruction of Nordt et al.^16^ (Supplementary Fig. 3 and Supplementary Table 2). This maximum value is broadly consistent with the upper end of fossil-leaf stomatal estimates for the T-OAE^8^ (Supplementary Text 1).

### Modelled carbon sources during the T-OAE

**Fig. 3:**
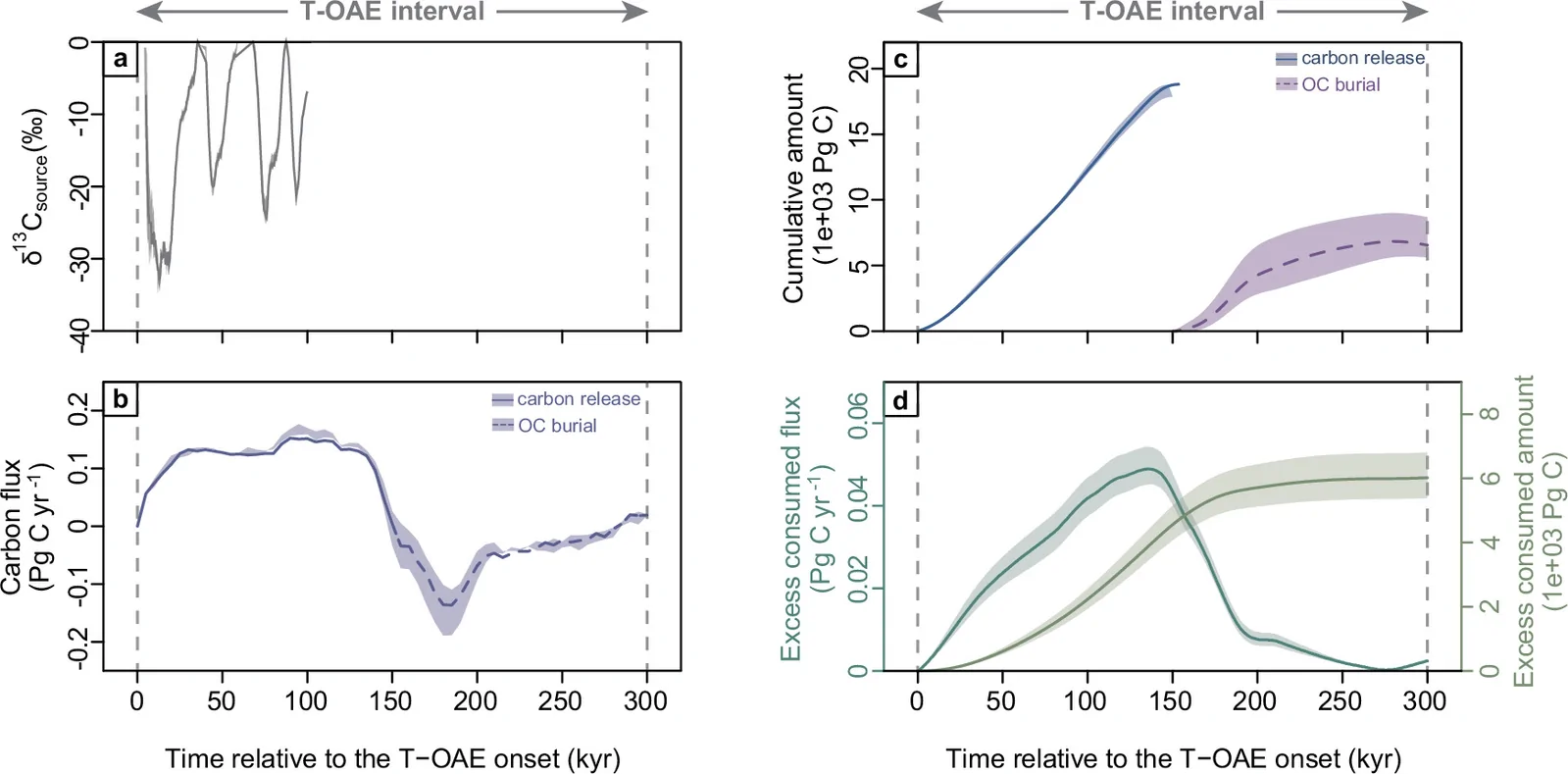
Model outputs during the T-OAE. **a** Modelled temporal evolution of δ^13^C_source_. **b** Modelled rates of carbon release (positive values, solid line) and OC burial (negative values, dashed line). **c** Modelled cumulative carbon release masses and OC burial masses. **d** Modelled rates and cumulative masses of excess carbon consumption due to enhanced continental silicate weathering. Shaded envelopes on each panel represent the results using the smoothed lower and upper *p*CO_2_ profiles (experiments 1 and 3; Supplementary Table 2). *T-OAE* Toarcian Oceanic Anoxic Event; *OC* organic carbon.

In the standard experiment (experiment 2), the modelled flux-weighted δ^13^C_source_ averages −13.6‰ (Fig. 3a). The pattern of this carbon release is characterised by four pulses. The initial and largest pulse occurs at the N-CIE onset and lasts ~35 kyr; δ^13^C_source_ declines to −33.0‰ and has a flux-weighted mean of −17.2‰. The subsequent three pulses (lasting ~32, 20, and 13 kyr, respectively) exhibit comparatively heavier isotopic compositions, with minimum δ^13^C_source_ values of ~−23‰ and flux-weighted mean values of ~−11‰ (Fig. 3a).

Previously proposed carbon sources include mantle-derived carbon (δ^13^C = −6‰)^26^, thermogenic carbon (−35‰ < δ^13^C < −20‰)^27^, and biogenic methane (δ^13^C = −60‰)^27^. Our modelled average δ^13^C_source_ of −13.6‰ during the T-OAE therefore indicates that volcanic degassing dominated the overall carbon release through the event. This value is close to that inferred for the End-Permian mass extinction (~252 Ma; mean δ^13^C_source_ = −15‰)^28^ and the Palaeocene–Eocene Thermal Maximum (~56 Ma; mean δ^13^C_source_ = −11‰)^29^, both of which were characterised by massive carbon release and pronounced global warming^30^. The convergence of δ^13^C_source_ estimates across these hyperthermals points to a common, volcanogenic origin of carbon, highlighting the pivotal role of deep-Earth processes in driving major perturbations of Earth’s climate^31^.

The lighter isotopic signature during the first pulse suggests a substantial contribution from a carbon source more depleted in ^13^C than volcanogenic carbon, possibly thermogenic carbon or equal contributions from biogenic and mantle-derived carbon. Indeed, the release of biogenic carbon from a climate-sensitive reservoir would be consistent with the astronomical periodicity in δ^13^C changes through the N-CIE onset^9–11^. The subsequent three pulses, characterised by higher minimum δ^13^C_source_ values, are best explained by episodic release of thermogenic and/or biogenic carbon superimposed on sustained volcanic degassing.

Across the different *p*CO_2_ forcing scenarios we consider (experiments 1–4), the flux-weighted mean δ^13^C_source_ remains largely stable, ranging from −13.7‰ to −10.7‰ (Supplementary Table 2). The maximum-*p*CO_2_ scenario (experiment 4; Supplementary Fig. 3) yields the highest value of −10.7‰. Overall, all four scenarios preserve a multi-pulse structure of carbon input and require a dominant contribution from relatively ^13^C-enriched magmatic carbon. Thus, the temporal pattern of release and the carbon source interpretation are broadly robust to the uncertainties in *p*CO_2_ across the T-OAE.

Uncertainty in our radioisotopic age model (± 119 kyr)^17^ may affect the temporal correspondence between the minimum δ^13^C_atm_ and peak *p*CO_2_. Sensitivity experiments exploring alternative age alignments (experiments 5–7; see “Methods”) yield systematically lighter carbon-source signatures (flux-weighted means of −21.7‰ to −25.1‰; Supplementary Fig. 4c), with minimum values reaching ~−60‰ at the onset of the event. These results suggest that greater contributions from isotopically light carbon reservoirs, such as thermogenic carbon and/or biogenic methane, cannot be excluded under alternative age-model assumptions. Nevertheless, the overall temporal pattern of carbon release remains broadly similar to that of the standard experiment, preserving the characteristic multi-pulse structure (Supplementary Fig. 4c). Because the standard experiment is based on the currently best-constrained age model^17^, our preferred interpretation remains that mantle-derived carbon constituted the dominant source of carbon release during the T-OAE. However, improved geochronological constraints will be important for further evaluating the contributions of thermogenic carbon and biogenic methane.

Karoo–Ferrar magmatism is widely regarded as the primary driver of the T-OAE^32^, and our model-diagnosed carbon sources are consistent with this interpretation. High-precision U–Pb geochronology demonstrates a close temporal correspondence between Ferrar LIP magmatism and the T-OAE^7,17,33^. The main phase of Karoo volcanism slightly predates the event, though magmatic activity likely continued through the T-OAE^34^. Elevated sedimentary Hg concentrations and excess Hg loading during the T-OAE further support intensified magmatic activity and increased volcanic inputs to the Earth’s surface system^35–37^. In addition, coupled Ca–C isotope mass-balance calculations, which integrated enhanced continental weathering fluxes with carbon-isotope constraints, suggested a δ^13^C_source_ of approximately −14‰ to −8‰ during the T-OAE^38^, consistent with a dominant volcanic CO_2_ contribution and our model results. Nevertheless, previous work has emphasised that volcanic degassing alone was unlikely to account for the full magnitude of the N-CIE because this would invoke unrealistically large carbon release that conflicts with proxy-based *p*CO_2_ and SST changes^39^. Consequently, thermogenic carbon release associated with magma intrusion into OC-rich sediments has been proposed as an additional driver^8,22^. Our recognition of pulses of carbon release during the N-CIE onset with δ^13^C_source_ values demonstrably lower than the mantle value aligns with these conclusions. Indeed, our model results and sensitivity experiments also do not rule out a potential contribution from more depleted carbon reservoirs at the N-CIE onset, such as biogenic carbon release from methane hydrates on continental shelves^2,9^, soil permafrost^10^, and/or continental environments such as wetlands, lakes, and soils^11^.

### Modelled carbon-release rates and cumulative masses during the T-OAE

**Fig. 4:**
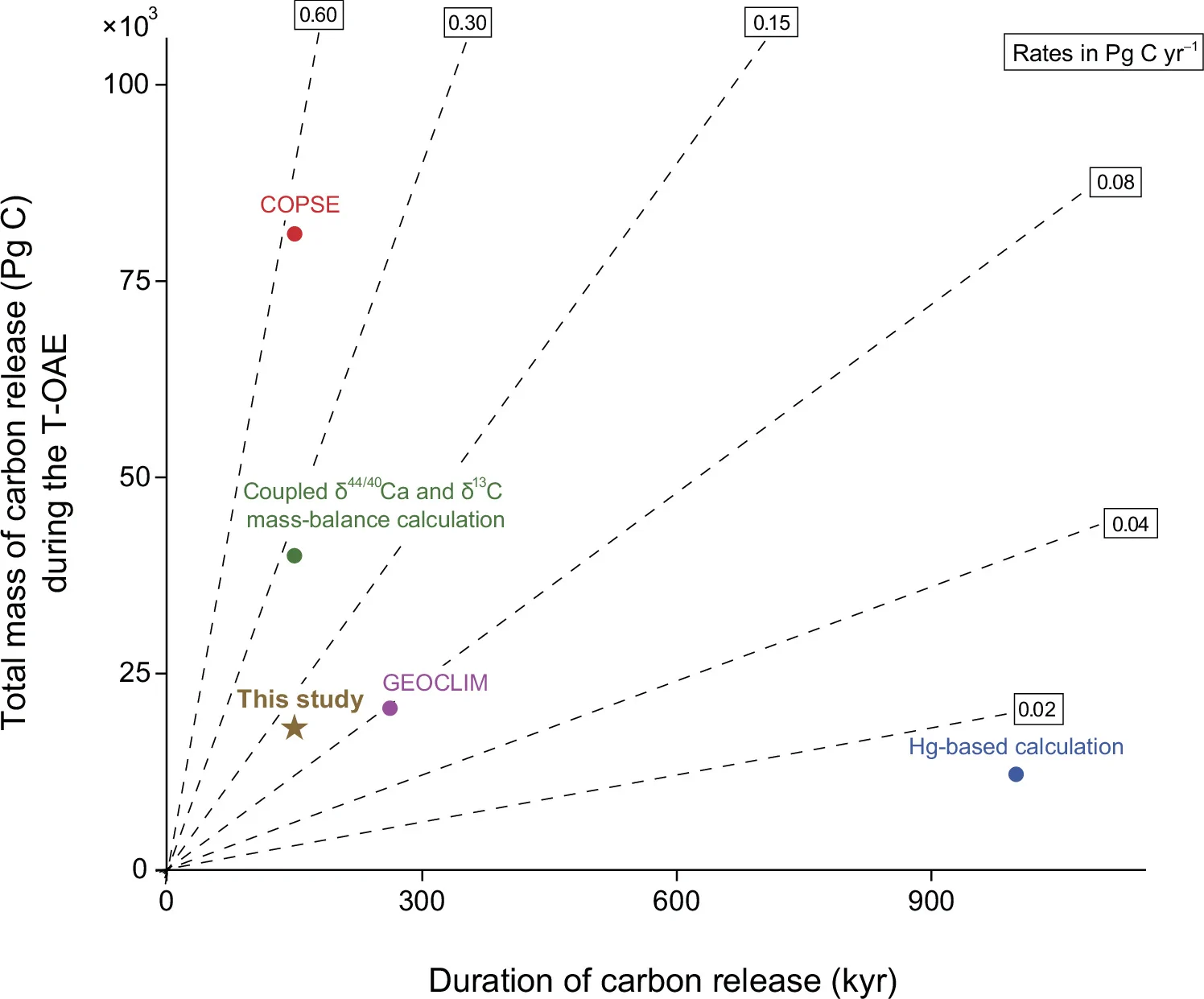
Comparison of published estimates of carbon release during the T-OAE with our cGENIE model results. Plot is modified from ref. ^37^ under a Creative Commons Attribution 4.0 International License. The brown star denotes the cGENIE-derived estimate of carbon release in the standard experiment (experiment 2). Published estimates from COPSE^20^, GEOCLIM^22^, coupled Ca–C isotope mass-balance calculations^38^, and Hg-based numerical modelling^37^ are also shown. Note that our estimate of the carbon release timescale is based on the interval of positive carbon flux identified by the model. *T-OAE* Toarcian Oceanic Anoxic Event.

Our standard experiment (experiment 2) yields a peak carbon-release rate of 0.16 Pg C yr^−1^, a mean flux of 0.12 Pg C yr^−1^, and a cumulative release of ~18,000 Pg C (Fig. 3b, c; Supplementary Table 2). Under the maximum-*p*CO_2_ forcing used in experiment 4, the peak release rate reaches 0.21 Pg C yr^−1^ and cumulative release reaches ~26,000 Pg C (Supplementary Fig. 3 and Supplementary Table 2). These differences emphasise that inferred carbon-release rates and cumulative masses are sensitive to the magnitude of the imposed *p*CO_2_ forcing. Nevertheless, the release rates and masses we obtain across our experiments fall within the range reported by previous modelling work on Karoo–Ferrar associated carbon release, including coupled Ca–C isotope mass-balance calculations (0.27 Pg C yr^−1^)^38^, GEOCLIM (0.08 Pg C yr^−1^)^22^, and COPSE (0.54 Pg C yr^−1^)^20^. Our inferred cumulative release of ~18,000 Pg C is much lower than the COPSE-based estimate (81,000 Pg C)^20^, but broadly comparable to the results derived from GEOCLIM (20,500 Pg C)^22^ and coupled Ca–C isotope mass-balance modelling (40,000 Pg C)^38^ (Fig. 4).

Varying assumptions regarding the size of the pre-event exogenic carbon reservoirs (i.e., baseline *p*CO_2_) contribute to the differences among model results^20,22^. For example, the larger carbon release estimated by COPSE likely reflects the use of a high initial *p*CO_2_ (~560–1120 ppm)^20^ relative to both our cGENIE simulations (~300 ppm) and the GEOCLIM work (~500 ppm)^22^. In our study, the use of a lower baseline *p*CO_2_ is based on a recently developed high-resolution reconstruction, which provides improved temporal constraints on *p*CO_2_ evolution^16^. A relatively low pre-T-OAE *p*CO_2_ is also consistent with evidence for a short-lived cooling or glaciation episode ~100 kyr prior to T-OAE warming^16,40^. By contrast, the slightly higher initial *p*CO_2_ adopted in GEOCLIM^22^ reflects the limited availability of high-resolution proxy constraints at the time, which relied upon *p*CO_2_ estimates derived from low-resolution stomatal data with large chronological uncertainties^8^. The considerably higher baseline *p*CO_2_ used in COPSE^20^ was primarily intended to reproduce the magnitude of warming inferred from proxy records under generally warm Early Jurassic background conditions. However, this approach may be influenced by overestimation of proxy-derived SST (see discussion below) and may not fully account for evidence of short-lived cooling immediately preceding the T-OAE. Moreover, differences in the carbon-isotope records used to constrain carbon release may also contribute to the contrasting model outputs. For example, in the recent model of Qiu et al.^12^, the N-CIE derived from bulk organic carbon at the Yorkshire section (UK)^9^ was used to constrain global carbon release. However, changes in organic matter sources can amplify the apparent N-CIE magnitude^41^. This issue could explain the high carbon release calculated from this model (up to ~73,000 Pg C)^12^, especially given the systematically larger N-CIEs recorded in organic (5–7‰) relative to inorganic carbon reservoirs (2–4‰)^32^.

Local processes, such as diagenetic effects, may bias primary δ^13^C_carb_ records^42,43^, resulting in substantial inter-site variability and anomalously negative values (Supplementary Fig. 1a). This may bias our δ^13^C_atm_ record and thus potentially bias the estimates of carbon release. Nevertheless, statistical analysis of the T-OAE N-CIE magnitude from individual sections indicates a median value of 3.2‰ (Supplementary Fig. 1b), closely matching the 3‰ magnitude inferred from our smoothed δ^13^C_carb_ stack (Fig. 2b). As such, sections affected by local overprinting exert only limited influences on our δ^13^C_atm_ record. Indeed, our compilation of 38 separate δ^13^C_carb_ records reduces sensitivity to site-specific biases and outliers, providing a more representative constraint on perturbations to the exogenic carbon reservoir during the T-OAE. Similar multi-record compilation approaches have been widely applied in palaeoclimate and palaeoenvironmental reconstructions and are considered effective for extracting robust first-order signals from spatially heterogeneous datasets^44^. Such refinement is particularly important during warming intervals, when intensified OC remineralisation may introduce isotopically light dissolved inorganic carbon into subsurface waters, distorting local isotope records^45^. Smoothing of the reconstructed *p*CO_2_ record would attenuate its variability and thus lead to some underestimation of reconstructed carbon release, and specifically carbon-release rates (an effect evaluated through our sensitivity experiments; see “Methods”). However, the inclusion of interactive ocean circulation, sedimentation, and multi-element cycling in the intermediate-complexity cGENIE model (see “Methods”) allows a more comprehensive representation of Earth system feedbacks than earlier modelling work. Taken together, our simulations provide improved quantifications of both carbon-release rates and cumulative masses during the T-OAE.

### Geological and stratigraphic evidence for Karoo–Ferrar carbon release

Although geological constraints on carbon generation and release associated with the Karoo–Ferrar LIPs remain incomplete, particularly for the Ferrar province, where much of the intrusive system is concealed beneath the Antarctic ice sheet, several independent geological and stratigraphic observations provide useful context for evaluating the plausibility of the reconstructed carbon-release history. Recent Hg-based reconstructions^37^, derived from sedimentary Hg accumulation and assumed volcanogenic Hg/CO_2_ scaling relationships, suggested cumulative carbon release of ~12,000 Pg C associated with Karoo–Ferrar activity, with an inferred carbon flux of ~0.012 Pg C yr^−1^. This Hg-derived cumulative carbon release is slightly lower than but broadly comparable with our cGENIE-based estimate, whereas the inferred flux is substantially lower (Fig. 4). These differences partly reflect the longer T-OAE duration (1 Myr) adopted in the Hg-based reconstruction^37^, but may also reflect potential attenuation of Hg signals during long-distance atmospheric transport from southern Gondwana to the northern hemisphere mid-latitude depositional site (Wales, UK). More importantly, sedimentary Hg records primarily constrain volcanic input fluxes, whereas Earth-system models simulate the integrated system response, involving state-dependent climate and environmental feedbacks (e.g., LIP-driven climate change and associated greenhouse gas release) and may therefore imply larger carbon fluxes and cumulative masses^37^. This interpretation is supported by a recent study using another model that incorporates rapid biogenic methane (CH_4_) release to account for the stepwise pattern of the δ^13^C record during the T-OAE, resulting in substantially larger carbon fluxes (up to 1.51 Pg C yr^−1^) and cumulative emissions (up to 73,000 Pg C) when combined CO_2_ and CH_4_ inputs are considered^12^.

Independent magmatic constraints provide a complementary first-order estimate of volcanic carbon inventories. Using reconstructed Karoo–Ferrar magma volumes (~2.5 × 10^6^ km^3^)^46^ and plausible magmatic CO_2_ concentrations (~0.4–1.3 wt%) constrained by Deccan Traps (~66 Ma) melt-inclusion studies^47^, first-order calculations suggest a total magmatic carbon inventory of ~7400–23,900 Pg C associated with Karoo–Ferrar magmatism. Although this estimate represents contained magmatic carbon rather than the fraction ultimately degassed to the atmosphere, it places bounds on the magnitude of plausible volcanic carbon release associated with Karoo–Ferrar activity, and brackets our estimate of 18,000 Pg C released across the T-OAE as a whole.

Additional geological observations from Karoo–Ferrar intrusive systems (Supplementary Text 3) indicate prolonged and spatially variable intrusive activity, consistent with episodic volatile release associated with sill emplacement and contact metamorphism. Basin-scale reconstructions based on Karoo intrusion geometry, host-rock distribution, and devolatilisation processes further estimate thermogenic carbon generation of ~7500 Pg C during Karoo magmatism^48^. Although lower than our cGENIE-derived cumulative carbon release (~18,000 Pg C), this thermogenic estimate is comparable in magnitude and indicates that intrusion-driven devolatilisation could have constituted an important component of Karoo–Ferrar carbon release. At the same time, the difference between thermogenic carbon generation alone and total modelled release suggests that additional sources and feedbacks, including volcanic degassing and mobilisation of exogenic carbon reservoirs, were required to reproduce the full magnitude of the T-OAE carbon-cycle perturbations. Taken together, Hg-based reconstructions, first-order magma-carbon inventories, and thermogenic carbon-generation estimates provide independent constraints that are broadly compatible with our reconstructed carbon-release masses and fluxes. Although each approach is associated with substantial uncertainty (particularly given the limited geological constraints currently available for parts of the Karoo–Ferrar LIPs), none requires carbon-release magnitudes fundamentally different from those inferred by the cGENIE simulations.

### Modelled SST and seawater pH changes during the T-OAE

**Fig. 5:**
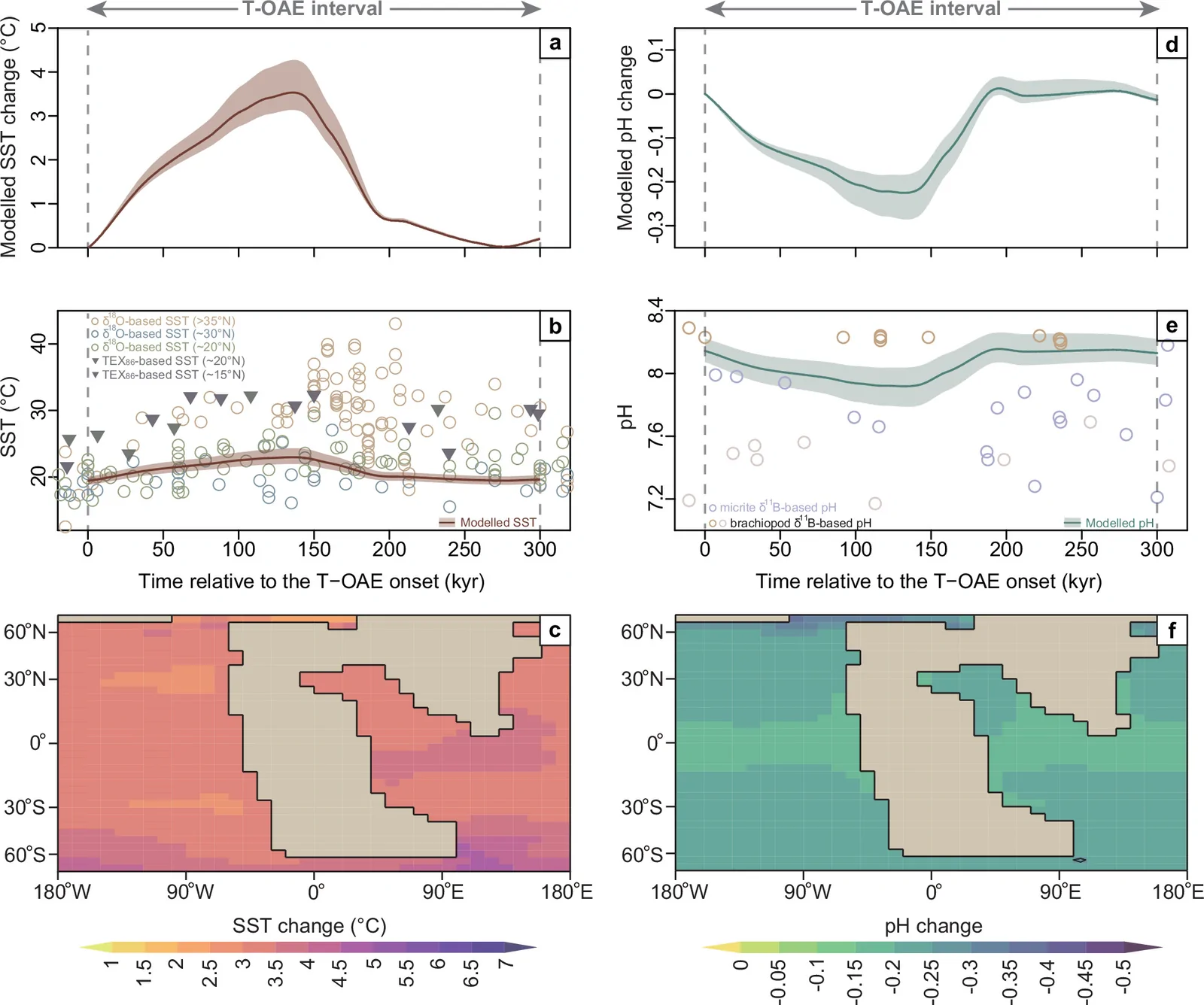
Model–proxy comparison and spatial patterns of SST and seawater pH changes across the T-OAE. **a** Modelled change in global average SST. The solid curve shows the result using the smoothed median *p*CO_2_ profile (experiment 2), and the shaded envelope shows the results using the smoothed lower and upper *p*CO_2_ profiles (experiments 1 and 3). **b** Comparison between model- and proxy-based SST reconstructions. Biomarker records (TEX_86_) are from ref. ^51^, and δ^18^O data (fossil shell carbonate) are from ref. ^10^ and references therein. Proxy data are grouped as a function of palaeolatitude^10,51^. **c** Spatial distribution of modelled SST change using the smoothed median *p*CO_2_ profile (experiment 2). **d** Modelled change in global average seawater pH. The solid curve shows the result using the smoothed median *p*CO_2_ profile (experiment 2) and the shaded envelope shows the results using the smoothed lower and upper *p*CO_2_ profiles (experiments 1 and 3). **e** Comparison between model- and proxy-based pH reconstructions. Proxy data are based on δ^11^B of brachiopods and micrite^21,56^. A consistent pH calibration method is applied to all δ^11^B records (ref. ^88^; see “Methods”). **f** Spatial distribution of modelled surface-ocean pH change using the smoothed median *p*CO_2_ profile (experiment 2). Spatial patterns of modelled SST and seawater pH changes using the smoothed lower (experiment 1) and upper (experiment 3) *p*CO_2_ profiles are shown in Supplementary Fig. 5. *T-OAE* Toarcian Oceanic Anoxic Event; *SST* sea surface temperature.

Our simulations indicate that massive carbon release during the T-OAE led to a global average SST increase of ~4 °C (Fig. 5a). Importantly, and in contrast to previous carbon-cycle studies based on box or reduced-complexity models, the cGENIE framework resolves the spatial structure of climate and ocean responses, revealing pronounced latitudinal heterogeneity in both SST and seawater pH changes (Fig. 5c, f). This spatially explicit perspective provides additional, testable constraints on the dynamics of the T-OAE and highlights the potential role of regional oceanographic processes. For example, in experiment 2, SST increases are relatively muted in tropical/subtropical regions (~3 °C) but substantially amplified at higher latitudes, reaching 5–6 °C in the Antarctic realm (Fig. 5c). This pattern is broadly consistent with previous results from fully coupled ocean–atmosphere simulations, which likewise identified modest low-latitude warming and amplified polar temperature increases of up to ~8 °C during the T-OAE^49^. Such high-latitude amplification has been attributed to intensified hydrological cycling, surface freshening, enhanced stratification, and weakened overturning circulation^49^ – mechanisms that are also included in our coupled Earth system simulations. Furthermore, δ^18^O-based temperature reconstructions independently show a similar spatial pattern (Fig. 5b). Based on the palaeogeographic distribution of compiled biocalcifiers^10^, δ^18^O-based SST estimates indicate lower magnitude warming in low- to mid-latitudes (20–30°N) and more pronounced increases at higher latitudes (>35°N). Notably, the magnitude of δ^18^O-inferred warming is generally larger (5–7 °C), and in some high-latitude sections even exceeds 10 °C, relative to our model-based SST increase (~4 °C) (Fig. 5b). Such discrepancies are expected, as many δ^18^O records–particularly those from the European Epicontinental Shelf in proximity to the landmass–are likely influenced by local hydrography (e.g., paleosalinity changes)^18^. In addition, belemnites, as nektonic species, may integrate temperatures over a range of water depths, and their δ^18^O signatures can be further modified by complex vital effects^50^, complicating direct interpretation of absolute SST changes across the T-OAE^18^.

A larger increase in SST (~10 °C) has also been inferred from TEX_86_ biomarker records in the tropical western Tethys at palaeolatitudes of ~15–20°N (Fig. 5b)^51^. However, sedimentary TEX_86_ values are known to be sensitive to nutrient availability, with oligotrophic conditions typically yielding elevated TEX_86_-derived temperatures^52^. This might be the case for the T-OAE, as weakened meridional overturning circulation during this interval would have disrupted nutrient supply pathways that were largely sustained by trade wind-driven upwelling in tropical regions^49^. The inferred collapse of nutrient supply is supported by a decline of nitrogen isotopes in the Ratnek El Kahla section (Algeria) at the northern margin of Gondwana (~20°N) across the T-OAE, which indicates a depletion of nitrate^53^. Under nutrient-stressed conditions, elevated TEX_86_ values would therefore be expected^52^, suggesting that TEX_86_-based SST reconstructions for the T-OAE may, at least in part, overestimate actual surface warming. Conversely, given the relatively low equilibrium climate sensitivity adopted in our cGENIE simulations (2.4 °C per doubling of *p*CO_2_, compared with 2.5–4 °C in IPCC AR6^54^), our modelled SST increase likely represents a lower-bound estimate. Together, these considerations highlight the importance of integrating proxy-based reconstructions with physically based models to robustly constrain the magnitude of T-OAE warming.

Ocean acidification associated with massive carbon release during the T-OAE has been widely invoked to explain reduced CaCO_3_ burial and coeval marine ecological catastrophe^55^. This interpretation is supported by seawater pH reconstructions based on boron isotopes (δ^11^B) from brachiopod shells and micrites in Europe, NW Tethys, which suggest a pH decline of ~0.3–0.5 units across the event^21,56^. Our modelled results show a decline of seawater pH by 0.2–0.3 units during the T-OAE (Fig. 5d), which is broadly comparable to the δ^11^B-based estimates (Fig. 5e), though the proxy-based pH quantification remains largely uncertain due to the physiological buffering capacity of the analysed taxa, potential detrital contamination, and lack of accurate micrite-based pH calibration^21^. Our simulations further resolve a latitudinally heterogeneous pH response, with more pronounced acidification at high latitudes than at low latitudes (Fig. 5f). This pattern is analogous to that inferred for the End-Permian mass extinction, albeit with a steeper pH decline during the End-Permian owing to substantially larger carbon-release rates and masses^57^.

### Modelled carbon sequestration during the T-OAE

On geological timescales, excess carbon released into the ocean–atmosphere system can be sequestered primarily through enhanced OC burial and continental silicate weathering^58^. In experiment 2, negative carbon fluxes (reaching a minimum of −0.14 Pg C yr^−1^) reflect carbon sequestration in the form of ^13^C-depleted organic carbon (Fig. 3b). Integrated over the negative-flux interval, the model yields a total OC burial of ~6800 Pg C (Fig. 3c).

Owing to the inversion-based configuration of cGENIE, negative carbon fluxes and the associated OC burial are only invoked when carbon release can no longer reproduce the proxy constraints (i.e., during the N-CIE recovery interval) (e.g., ref. ^29^). As a result, OC burial during the N-CIE onset interval is not explicitly captured in our simulations, and the modelled burial therefore likely represents a lower-bound estimate for the entire event. A compilation of total organic carbon (TOC) records (*n* = 3832) from 64 globally distributed marine sections (see “Methods”) shows that, although TOC begins to increase near the N-CIE onset, maximum enrichment is not reached until ~180 kyr after the initiation of the excursion, coincident with the N-CIE recovery phase (Supplementary Fig. 6). A detailed analysis of these data (see “Methods”) suggests that ~30% of the total OC buried during the event may have occurred during the onset interval, with the remaining ~70% buried during the recovery interval. Applying this partitioning estimate to the published whole-event estimate of 9000 Pg C of net additional OC burial^13^ suggests that ~6300 Pg C was buried during the recovery–close to the ~6800 Pg C quantified in experiment 2. Equally, if we assume that ~30% of OC burial (i.e., ~2700 Pg C) is not captured by the cGENIE model during the N-CIE onset, then it would imply a higher cumulative release of ~20,500 Pg C. Assuming a buried OC δ^13^C value of −27‰^59^ shifts the whole-event flux-weighted δ^13^C_source_ from −13.6‰ to −15.4‰ (see “Methods”). Thus, omission of the onset interval OC burial likely causes an underestimation of cumulative carbon release and requires a moderately lighter carbon source composition.

Marine alkalinity flux could have increased in response to the warming-enhanced continental weathering, through which more Ca^2+^ and bicarbonate (HCO_3_^-^) ions were generated and transported to the oceans^60^. In contrast to carbonate weathering, silicate weathering would cause net CO_2_ consumption (e.g., ref. ^14^). Assuming a constant ratio of carbonate-to-silicate weathering during the T-OAE (e.g., ref. ^61^), we quantify excess carbon consumption by evaluating the relative change in silicate weathering-driven Ca^2+^ fluxes between pre- and syn-T-OAE conditions (see “Methods”). Our simulations (experiment 2) yield an excess carbon consumption rate peaking at ~0.05 Pg C yr^−1^ and a cumulative carbon uptake of ~6000 Pg C through the event (Fig. 3d). This enhanced weathering sink, acting in concert with increased OC burial, likely played an important role in facilitating the recovery of the perturbed Earth system.

Expansion of marine anoxia during the T-OAE would also have promoted the accumulation of dissolved organic carbon (e.g., ref. ^62^), while enhanced alkalinity delivery and carbonate compensation could have increased carbon storage within the oceanic dissolved inorganic carbon reservoir (e.g., ref. ^63^). Neither process is explicitly quantified here, but both may have contributed to carbon sequestration during the event and may partly explain the difference between cumulative carbon release and the combined effects of modelled OC burial and silicate weathering consumption. Quantifying the size and longevity of these oceanic carbon reservoirs warrants further investigation.

Overall, our cGENIE-based results provide an improved quantitative estimate of how the global carbon cycle may have evolved during the T-OAE owing to the complexity of the model, the parameterisation of a high-resolution *p*CO_2_ record, and the use of a compiled, globally representative T-OAE N-CIE with a high-precision age framework. At the same time, however, our smoothed C_3_ plant-derived *p*CO_2_ curve is a necessarily simplified quantification of the pattern of *p*CO_2_ change during the T-OAE, and does not readily capture very short-term changes in *p*CO_2_ that may have occurred through the event. As such, although our modelling approach provides a robust way of estimating carbon-release sources, rates, and masses as well as carbon sequestration during the T-OAE, the precise timing of these two processes could be improved by more detailed future work, such as more precise *p*CO_2_ reconstruction based on multiple proxies and the use of higher-resolution δ^11^B from material with limited vital effects and well-established calibrations for seawater pH reconstruction. Refining the model representation of continental weathering beyond its current dependence only on mean continental surface air temperature^29^, along with improved constraints on the relative roles of carbonate and silicate weathering, will strengthen quantitative reconstructions of the T-OAE carbon cycle.

### Implications for modern global warming

The peak carbon-release rate for the T-OAE (0.16 Pg C yr^−1^) is nearly two orders of magnitude lower than the modern anthropogenic release rate (~11 Pg C yr^−1^)^64^. Although the timescales being compared are vastly different, our new findings contribute to an emerging view that hyperthermal events in Earth history were unlikely to have involved release rates greater than those currently occurring through human activity. For example, previous modelling work determined a peak carbon-release rate of ~5 Pg C yr^−1^ during the End-Permian mass extinction^28^, an interval marked by extreme warming and severe biotic loss^30^. By contrast, substantially lower release rates (quantified on broadly similar timescales) have been inferred for OAE 1a (~0.01–0.7 Pg C yr^−1^)^25^ and the Palaeocene–Eocene Thermal Maximum (~0.6–1.7 Pg C yr^−1^)^29,58,65^, events that were associated with relatively more limited biotic turnover^30^. As such, our findings further underline how rates of carbon release, and the resulting pace of climate and environmental changes (e.g., temperature, pH, and redox), could have played a major role in governing ecosystem stability and biodiversity through Earth’s history. Indeed, this inference has been supported by more recent research^66,67^, which indicates that rapid rates of seawater temperature and pH changes, rather than absolute background states, are most strongly associated with major extinction events in the Phanerozoic. Viewed in this context, the exceptionally rapid rate of modern anthropogenic carbon release represents a fundamental departure from the tempo of past hyperthermal events, with the potential to overwhelm stabilising Earth system feedbacks and drive environmental and biological responses beyond those documented in the geological record.

## Methods

### Duration of the T-OAE

The age model for our simulations is based on the latest high-precision U–Pb radioisotopic dating from three tuff horizons spanning the T-OAE in the Sakuraguchi-dani section, Japan. This work determined a T-OAE (N-CIE) duration of ~300 kyr, with the onset at ~182.875 Ma^17^. This estimate is in broad agreement with some previous cyclostratigraphic constraints^68–70^. Specifically, a 405 kyr tuned cyclostratigraphic analysis of upper Rhaetian to lower Toarcian chert bed thickness data in the Katsuyama-Sakahogi composite section, Japan, determined a T-OAE duration of ~260 kyr^68^. This is close to similar cyclostratigraphic analyses of European and Moroccan sections, which have yielded durations of 300–500 kyr for the T-OAE^69,70^.

### T-OAE N-CIE magnitude

To remove high-frequency variability likely resulting from analytical error and local factors, a LOESS fit method with 0.5 kyr spacing was used to smooth the stacked δ^13^C_carb_ records to reveal the overall trends (Fig. 2b). Notably, a key parameter in LOESS is the span, which determines the fraction of the data used for each local fit. Typically, a smaller span value (e.g., 0.01) captures finer-scale variations but may overfit local noise, while a larger span (e.g., 0.99) results in stronger smoothing, potentially obscuring important short-term signals. To balance the suppression of local noise with the preservation of rapid geochemical changes, previous studies often adopted span values ranging from 0.1 to 0.3 (e.g., ref. ^71^). In this study, we systematically tested a broad range of span values from 0.01 to 0.99 for the input δ^13^C_atm_ and *p*CO_2_ records. For each span, we calculated the fitting error, quantified as the mean squared error between the LOESS-smoothed curve and the original dataset. As shown in Supplementary Fig. 7a, the fitting error increases with span across the δ^13^C_atm_ dataset, indicating that broader smoothing leads to greater deviation from the raw data. Notably, a span value of ~0.1 marks a threshold beyond which errors begin to increase sharply. Based on this trend, we selected a span value of 0.1 for the LOESS fitting of the stacked δ^13^C_atm_ records, as it effectively reduces local noise while retaining the intrinsic variability and structure of the dataset. Despite some fluctuations, our smoothed δ^13^C_atm_ profile shows an overall decreasing trend from −6.62‰ at the T-OAE onset (t = 0 kyr) onwards (Fig. 2b). This initial value is broadly comparable to the fossil-based δ^13^C_atm_ reconstructions^72^. Our fitted δ^13^C_atm_ data reach a minimum of −9.61‰ at 100 kyr, followed by a gradual recovery to the end of the T-OAE (−6.33‰ at 300 kyr).

The N-CIE magnitude (3‰) was calculated as the absolute difference between the fitted minimum δ^13^C_carb_ and the fitted value at 0 kyr. Although almost all compiled δ^13^C_carb_ profiles come from the Tethyan realm in the northern hemisphere (with only one site from Kuwait likely palaeogeographically close to the equator; site 38 in Fig. 1a), the fitted δ^13^C_atm_ (converted from δ^13^C_carb_) curve is expected to be globally representative because the N-CIE magnitude we calculated (3‰) is comparable to that recorded in δ^13^C_carb_ in sections from the southern hemisphere (e.g., 3‰ at El Peñon and 4‰ at Asientos in Chile, ref. ^73^; note these two δ^13^C_carb_ profiles were not included in our data compilation because the N-CIEs are stratigraphically incomplete at both sites). Our calculated N-CIE magnitude is also in agreement with the magnitude calculated from δ^13^C_org_ data that had been corrected for the impact of changing organic matter sources (3‰ to 4‰)^41^. Terrestrial long-chain *n*-alkanes recorded in Europe (Iberian Basin, Spain and Cleveland Basin, UK) and China (Sichuan Basin), with an average N-CIE magnitude ranging from 3.1‰ to 4.2‰^39,74,75^ also support our inference, though abnormally high values (5.2‰ to 11.3‰) occur in Junggar Basin and Ordos Basin (China) most likely due to local variability in vegetation and humidity in continental interiors^76,77^. In addition, brachiopod- and belemnite-based δ^13^C_atm_ reconstructions show the N-CIE magnitude of 2.2‰ to 3‰^72^, matching well our inference. Taken together, we infer that our calculated N-CIE magnitude of 3‰ represents a reasonable value for the magnitude of the event in the exogenic carbon cycle.

### Correlation between compiled δ^13^C_carb_ and *p*CO_2_

The *p*CO_2_ record used in our study is based on the C_3_ plant CO_2_ proxy, as quantified from fossil wood carbon isotope (δ^13^C_wood_) data from the Peniche section (Portugal) by Nordt et al.^16^. The Peniche section has also yielded a high-resolution carbon-isotope record from carbonates^3^, thereby allowing for the robust alignment of the reconstructed *p*CO_2_ with our high-resolution stacked δ^13^C_carb_ record on the same timescale (Fig. 2a, b). In detail, based on the composite δ^13^C_wood_ and δ^13^C_carb_ morphology in the Peniche section^3^, we set section heights of 1045 cm and 3061 cm as the onset and end of the T-OAE N-CIE, respectively. Given a T-OAE N-CIE duration of 300 kyr^17^, the average sedimentation rate in the Peniche composite δ^13^C_wood_ and δ^13^C_carb_ profile is 6.72 cm kyr^−1^. Using this estimated sedimentation rate, we obtained the age of each δ^13^C and *p*CO_2_ value (Fig. 2a, b). The *p*CO_2_ record (including lower, median, and upper profiles defined by Nordt et al.^16^) was then smoothed using a LOESS fit method with 0.5 kyr spacing (Fig. 2a). Given an abrupt increase in errors when span values increase from 0 to 0.2 (Supplementary Fig. 7b), we instead used the Generalised Cross-Validation method (previously used for the cGENIE modelling work during the End-Permian mass extinction)^57^ in package *fANCOVA* via *R* to determine the best degree of smoothing for LOESS fitting of the input *p*CO_2_ records (Fig. 2a). Because smoothing of the input *p*CO_2_ record inevitably attenuates short-lived extremes, we additionally constructed an alternative *p*CO_2_ curve based on the upper envelope of the proxy-derived *p*CO_2_ reconstruction from Nordt et al.^16^, which reaches a maximum of ~1750 ppm during the T-OAE. This record was linearly interpolated onto the same age framework as the δ^13^C_atm_ record (Supplementary Fig. 3a, b), thereby retaining the full amplitude of reconstructed *p*CO_2_ variability while maintaining temporal consistency between the two forcing records.

### Earth system model

We use the cGENIE model (carbon-centric Grid-Enabled Integrated Earth system model) to quantify the carbon cycle during the T-OAE. cGENIE is an Earth system model of intermediate complexity, consisting of a three-dimensional (3D) ocean general circulation model, a 2D dynamic-thermodynamic sea-ice model, and a 2D energy-moisture balance model in the atmosphere^78^. The model accounts for the biogeochemical cycling of various elements and isotopes (e.g., carbon, sulphur, oxygen, and phosphorus) in the ocean^79^, plus the preservation and burial of carbonate in deep-sea sediments^63^ and a terrestrial weathering module^80^ to close the geological carbon cycle.

The cGENIE model we applied was configured on a 36 × 36 grid with 17 vertical levels in the ocean^81^. Previous studies commonly used a single-inversion approach, i.e., using δ^13^C records alone with a series of assumed carbon sources, to calculate the rate and cumulative mass of carbon release via the cGENIE model^24,28,58^. This would cause non-unique solutions of δ^13^C_source_, though the comparison between the model-based and the proxy-based results (e.g., SST and pH) could help narrow down the uncertainties and pick out the optimal carbon-cycle scenario^24,28,58^. Instead, a double-inversion approach, i.e., applying paired δ^13^C_atm_ and *p*CO_2_ (or seawater pH) to cGENIE, would yield a unique solution of δ^13^C_source_, carbon-release flux and mass^25,29,57^.

Given the physiological effect of the analysed taxa, potential detrital contamination, and inaccurate pH calibration of micrite-derived δ^11^B, reconstructed seawater pH based on δ^11^B from brachiopods and micrite^21^ was not used here to force the model. Alternatively, we adopted a double-inversion approach for the simulations with paired δ^13^C_atm_ and *p*CO_2_ data to decipher the global carbon cycle during the T-OAE. The palaeogeographic information and related boundary conditions, including the palaeobathymetry, wind fields, and planetary albedo used for our simulations are those of Pohl et al.^81^ for 180 Ma, derived from a general circulation model simulation using the palaeogeographic reconstruction of the PALEOMAP project^82^. We assume no significant variations of palaeogeography and boundary conditions for our T-OAE simulations, given the temporal proximity between the selected time slice (180 Ma) and the T-OAE (183 Ma).

We first spun up the model (20 kyr) in a closed system (i.e., no gain or loss of tracers from weathering or sedimentation), with a pre-event background *p*CO_2_ value of ~300 ppm. This *p*CO_2_ value falls within the range inferred from both stomatal index (350 ± 105 ppm)^8^ and the CO_2_ effect of C_3_ plants (225–314 ppm)^16^. Subsequently, the model was run as an open system for 200 kyr to reach an equilibrated state between carbon sources (volcanic outgassing) and sinks (silicate weathering, through carbonate burial in marine sediments). For the following simulations, the time step for the double-inversion approach was set at 0.5 kyr. At each time step, cGENIE adjusts the mass of emitted carbon into the atmosphere to reproduce the prescribed *p*CO_2_. On this basis, the model yields the required isotopic composition of the added carbon to reproduce the N-CIE. We set a maximum carbon flux of 10 Pg C yr^−1^ (close to the modern anthropogenic value)^64^ with δ^13^C_source_ ranging from −60‰ to 0‰ for the simulations to obtain the realistic outputs. Following Gutjahr et al.^29^, organic carbon burial is invoked and represented in the form of negative emissions when it is required to match the *p*CO_2_ curve used for the inversion.

### Sensitivity experiments

Sensitivity experiments were performed to evaluate the effects of uncertainties in *p*CO_2_ reconstruction and age alignment between *p*CO_2_ and δ^13^C_atm_. The lower and upper *p*CO_2_ profiles from Nordt et al.^16^ were smoothed and tested first (experiments 1 and 3, respectively; Supplementary Table 2). To evaluate the influence of potential underestimation of peak *p*CO_2_ arising from the smoothing procedure on modelled carbon release, an additional simulation was conducted using the alternative maximum *p*CO_2_ curve described above, which reaches ~1750 ppm during the T-OAE (experiment 4; Supplementary Fig. 3a). In addition, sensitivity experiments on age alignment between *p*CO_2_ and δ^13^C_atm_ were performed to evaluate the effects of chronological uncertainty (± 119 kyr; ref. ^17^) (experiments 5–7; Supplementary Table 2). In the standard experiment (experiment 2), the peak *p*CO_2_ occurs 35 kyr later than the minimum δ^13^C_atm_ (Fig. 2a, b). Given the dating uncertainty and the unlikelihood that maximum *p*CO_2_ preceded the minimum δ^13^C_atm_^57^, we applied a forward temporal shift of 35 kyr to the fitted *p*CO_2_ record to synchronise its maximum with the corresponding minimum δ^13^C_atm_ (phase alignment at 100 kyr; Supplementary Fig. 4a, b). Complete time-series outputs from the maximum-*p*CO_2_ and alternative age-alignment experiments are presented in Supplementary Figs. 3 and 4 and summarised in Supplementary Table 2. Additional carbon sequestration and spatial model outputs are discussed in Supplementary Text 2.

### Modelled silicate weathering-driven excess carbon consumption

The cGENIE model incorporates a dedicated rock weathering module (the Rock Geochemical Model)^80^, in which Ca^2+^ and alkalinity fluxes respond dynamically to climate-driven changes in land temperature. This module explicitly accounts for both carbonate and silicate weathering processes, represented by Eqs. (1) and (2), respectively, with each weathering regime parameterised through distinct geochemical formulations.

1 $${{{{\rm{CO}}}}}_{2({{{\rm{aq}}}})}+{{{{\rm{H}}}}}_{2}{{{{\rm{O}}}}}_{({{{\rm{l}}}})}+{{{{\rm{CaCO}}}}}_{3({{{\rm{s}}}})}\to {{{{{\rm{Ca}}}}}^{2+}_{({{{\rm{aq}}}})}}+{{2{{{\rm{HCO}}}}}^{-}_{3({{{\rm{aq}}}})}}$$

2 $${2{{{\rm{CO}}}}}_{2({{{\rm{aq}}}})}+{{{{\rm{H}}}}}_{2}{{{{\rm{O}}}}}_{({{{\rm{l}}}})}+{{{{\rm{CaSiO}}}}}_{3({{{\rm{s}}}})}\to {{{{{\rm{Ca}}}}}^{2+}_{({{{\rm{aq}}}})}}+{{2{{{\rm{HCO}}}}}^{-}_{3({{{\rm{aq}}}})}}+{{{{\rm{SiO}}}}}_{2({{{\rm{aq}}}})}$$

As illustrated by these equations, weathering of both carbonate and silicate rocks would increase seawater alkalinity, whereas only silicate weathering causes the net consumption of atmospheric CO_2_ on geological timescales.

On this basis, relative changes in modelled Ca^2+^ fluxes between pre- and syn-T-OAE conditions are interpreted as reflecting variations in continental weathering intensity and associated alkalinity delivery to the ocean. Adopting a carbonate-to-silicate weathering ratio of 0.6:0.4 (in terms of Ca^2+^ fluxes to the ocean) based on modern continental weathering analyses^83–85^ and its extension to deep-time systems^61^, we quantify the rates and masses of silicate weathering-driven excess carbon consumption during the T-OAE (Fig. 3d and Supplementary Fig. 4f).

### Compilation of TOC records and thickness-weighted partitioning of net OC burial

TOC data were taken from the compilation of Kemp et al.^13^. The continental records Anya, Sichuan Core A and Sichuan Core B were excluded. The final compilation comprised 3832 data points from 64 marine sections. Relative TOC enrichment (hereafter referred to as TOC anomalies) was defined as

3 $${\Delta {{{\rm{TOC}}}}}_{i}({{t}})={{{{\rm{TOC}}}}}_{i}({{t}})-{\overline{{{{\rm{TOC}}}}}}_{i,{{{\rm{pre}}}}}$$

where ${\overline{{\mbox{TOC}}}}_{i,{\mbox{pre}}}$ is the mean TOC between −50 and 0 kyr relative to the T-OAE N-CIE onset (based on extrapolation of sedimentation rates assuming a 300 kyr N-CIE duration in each of the records). The normalised records were then stacked on the same age framework as the δ^13^C_atm_ dataset. A LOESS fit method (span = 0.1) was then applied to characterise first-order temporal changes in relative TOC enrichment across the T-OAE (Supplementary Fig. 6).

Of the 64 marine sections, 47 have complete (i.e., untruncated) N-CIEs in Kemp et al.^13^. Records with only approximate (~x m) or lower-bound (>x m) thicknesses (see Table 1 in ref. ^13^) were excluded because they do not allow robust calculation of mean sedimentation rate. Belmont, Nianduo and Rabaçal were also excluded because their TOC records do not extend to 300 kyr. Each of the remaining 44 records was linearly interpolated onto a 0.1 kyr grid. TOC anomalies were then integrated over the N-CIE onset (0–100 kyr) and recovery (100–300 kyr) intervals using the following equations:

4 $${{{I}}}_{{{{\rm{on}}}},i}=\int ^{100}_{0}{\Delta {{{\rm{TOC}}}}}_{i}({{t}})\,{{dt}}$$

5 $${{{I}}}_{{{{\rm{rec}}}},i}=\int ^{300}_{100}{\Delta {{{\rm{TOC}}}}}_{i}({{t}})\,{{dt}}$$

Both positive and negative anomalies were retained because the 9000 Pg C estimate used for scaling represents net additional OC burial relative to background conditions^13^.

For each section, the mean sedimentation rate (${R}_{i}$) was estimated from its N-CIE thickness ${H}_{i}$:

6 $${{{R}}}_{i}=\frac{{{{H}}}_{i}}{{{{\rm{300\,kyr}}}}}$$

The onset and recovery integrals were then weighted by sedimentation rate:

7 $${{{W}}}_{{{{\rm{on}}}},i}={{{R}}}_{i}{{{I}}}_{{{{\rm{on}}}},i}$$

8 $${{{W}}}_{{{{\rm{rec}}}},i}={{{R}}}_{i}{{{I}}}_{{{{\rm{rec}}}},i}$$

The aggregate onset fraction across all sections was calculated from the summed onset and recovery weights as

9 $${f}_{{{{\rm{on}}}}}=\frac{{\sum }_{i=1}^{n}{{{W}}}_{{{{\rm{on}}}},i}}{{\sum }_{i=1}^{n}{{{W}}}_{{{{\rm{on}}}},i}+{\sum }_{i=1}^{n}{{{W}}}_{{{{\rm{rec}}}},i}}$$

where i denotes an individual section and *n* = 44. The recovery fraction was calculated as ${f}_{{{\mathrm{rec}}}}=1-{f}_{{{\mathrm{on}}}}$. This weighting approximates integration with respect to deposited stratigraphic thickness under the assumption of a constant mean sedimentation rate within each section.

Phase-specific burial masses were obtained by applying the estimated fractions to the published whole-event net burial estimate:

10 $${{{B}}}_{{{{\rm{on}}}}}=({{{\rm{9000\,Pg}}}}\,{{{\rm{C}}}}){f}_{{{{\rm{on}}}}}$$

11 $${{{B}}}_{{{{\rm{rec}}}}}=({{{\rm{9000\,Pg}}}}\,{{{\rm{C}}}}){f}_{{{{\rm{rec}}}}}$$

The potential effect of omitted onset burial on gross carbon input was evaluated using a first-order mass-balance assumption. Because onset OC burial removes carbon from the exogenic carbon reservoir, reproducing the same modelled carbon-cycle trajectory would require an equal amount of additional carbon release. The corrected cumulative release was therefore calculated as

12 $${{{M}}}_{{{{\rm{corr}}}}}={{{M}}}_{{{{\rm{model}}}}}+{{{B}}}_{{{{\rm{on}}}}}$$

where *M*_model _= 17,849 Pg C. The corresponding interval-average compensating flux was calculated as $\textstyle \bar{F}_{{\mathrm{on}}}=\frac{B_{{\mathrm{on}}}}{100\,{\mathrm{kyr}}}$, but was not added to the modelled peak release rate because the detailed timing of onset burial is unconstrained.

The corrected whole-event flux-weighted δ^13^C_source_ was then estimated using isotope mass balance:

13 $${{{\updelta}}}^{13}{{{{\rm{C}}}}}_{{{{\rm{source}}}},{{{\rm{corr}}}}}=\frac{{{{M}}}_{{{{\rm{model}}}}}{{{\updelta}}}^{13}{{{{\rm{C}}}}}_{{{{\rm{model}}}}}+{{{B}}}_{{{{\rm{on}}}}}{{{\updelta}}}^{13}{{{{\rm{C}}}}}_{{{{\rm{OC}}}}}}{{{{M}}}_{{{{\rm{model}}}}}+{{{B}}}_{{{{\rm{on}}}}}}$$

where δ^13^C_model_ was −13.6‰. We used δ^13^C_OC_ of −27‰ as a representative value for Phanerozoic black-shale organic matter^59^.

This analysis provides a temporal partition of a published whole-event burial estimate rather than an independent reconstruction of global OC accumulation. It does not account for variations in dry bulk density, compaction, depositional area, preservation, hiatuses, within-section sedimentation rates or age models. The corrected carbon-release mass and source composition are therefore reported as conditional sensitivities.

### Compilation of proxy-based SST and pH reconstructions

Proxy-based SST reconstruction was determined from oxygen isotopes of abundant belemnite fossils (δ^18^O_bel_) distributed largely in Europe, western Tethys^10^. The equation used to calculate the SST is from ref. ^86^ assuming a seawater δ^18^O of −1.0‰. Based on the correlation between the well-defined T-OAE N-CIE and the abundant δ^18^O_bel_ data^10^, the δ^18^O_bel_-derived SST was placed on the same timescale as our simulations using the δ^13^C chemostratigraphy (Fig. 5b). In addition, TEX_86_ palaeotemperature measurements have been used to estimate SST variations during the T-OAE at the Valdorbia section (Italy, ~15°N) and the La Cerradura section (Spain, ~20°N) in the southern area of the northwest Tethys shelf^51^. These TEX_86_-derived SSTs were calculated based on the equation from ref. ^87^. Based on the correlation between the complete T-OAE N-CIEs and the TEX_86_ data at both sections^51^, we placed the TEX_86_-derived SST on the same timescale as our simulations (Fig. 5b).

Proxy-based seawater pH measurements have been determined from boron isotopes of brachiopods (δ^11^B_bra_) and micrite (δ^11^B_mic_) in Europe^21,56^. In detail, based on δ^11^B_bra_ records in the Peniche section, Lusitanian Basin (Portugal) and a δ^11^B-ocean pH calibration method from ref. ^88^, a decline in seawater pH of ~0.35 units during the T-OAE has been estimated (Fig. 5e)^56^. However, this work has been challenged more recently because of the potential detrital contamination and physiological buffering capacity of the analysed taxa at Peniche^21^. Instead, δ^11^B_mic_ records from the Lusitanian Basin (Portugal) and Iberian Basin (Spain) can potentially track trends in seawater pH, though no δ^11^B-pH calibration exists for micrite to calculate the absolute seawater pH values^21^. To make a comparison between δ^11^B_bra_- and δ^11^B_mic_-based seawater pH reconstruction, the pH calibration method from ref. ^88^ (that had been used on δ^11^B_bra_ records in ref. ^56^) has also been used on δ^11^B_mic_ for seawater pH calculation (Fig. 5e)^21^. Based on the correlation between the well-defined T-OAE N-CIE and the δ^11^B_bra_ and δ^11^B_mic_ data in the Lusitanian Basin^3,20,56^ and Iberian Basin^20,21^, we placed the δ^11^B-derived seawater pH on the same timescale as our simulations (Fig. 5e).

## Supplementary information

Supplementary Information

Transparent Peer Review file

## Data Availability

The compiled δ^13^C_carb_ data used in this study are available from the original sources listed in Supplementary Table 1. The TOC data are from Kemp et al.^13^, and the *p*CO_2_ data are from Nordt et al.^16^.

## Code availability

The cGENIE configuration files used to reproduce the simulations are available in the Zenodo repository^89^.
