# Supplementary material for: Massive release of predominantly magmatic carbon drove an Early Jurassic hyperthermal
Source: Nat Commun. 2026 Sep 18;17:10968. doi: 10.1038/s41467-026-77747-y (PMC13683373; doi:10.1038/s41467-026-77747-y)
Supplement: Supplementary file 1 [file 41467_2026_77747_MOESM1_ESM.pdf]

Supplementary Information for

Massive release of predominantly magmatic carbon drove an Early Jurassic hyperthermal

Wenhan Chen *et al.*

*Corresponding authors: David B. Kemp, davidkemp@cug.edu.cn
Ying Cui, cuiy@montclair.edu
Chao Li, chaoli@cdut.edu.cn

This file includes:

Supplementary Text
Supplementary Figures 1 to 7
Supplementary Tables 1 and 2
Supplementary References

Supplementary Text

1. Additional background information

T-OAE N-CIE magnitude

Variations in the source and thermal maturity of sedimentary organic matter can bias the N-CIE associated with T-OAE carbon release, resulting in systematically larger excursion magnitudes in the organic matter carbon than in the carbonate carbon counterpart[1]. Consequently, bulk organic carbon isotopes ($\delta^{13}C_{org}$) alone are not sufficiently robust to precisely trace perturbations of the global exogenic carbon reservoir, although numerous high-resolution records of the T-OAE N-CIE in organic carbon have been identified worldwide in both marine and terrestrial settings[2–4]. Alternatively, $\delta^{13}C$ of land plant-derived *n*-alkanes has been employed to reflect the atmospheric signal[4–8]. However, the number of available sites is limited (n = 5; Iberian Basin, Spain; Cleveland Basin, UK; Sichuan, Ordos, and Junggar basins, China). The large inter-site variability in N-CIE magnitudes (3‰ to 11‰)[4–8], likely driven by local controls such as vegetation type and hydroclimate (e.g., refs. 7,8), further hampers reconstruction of a globally representative T-OAE N-CIE. In addition, warming-induced enhancement of organic matter remineralization through intensified microbial metabolism may have released extra ^{13}C-depleted carbon into seawater, thereby modifying the isotopic compositions of marine dissolved inorganic carbon and further complicating carbonate $\delta^{13}C$-based estimates of the N-CIE linked to large-scale carbon release into the exogenic system[9]. Taken together, these limitations underline the need for a globally representative N-CIE record that robustly reflects carbon release to the ocean–atmosphere system, as a prerequisite for quantitatively resolving the global carbon cycle during the T-OAE.

Reconstructed pCO_2 records across the T-OAE

Stomata-based measurements of fossil leaves from the Bornholm, Danish Basin (Denmark) indicated that atmospheric pCO_2 increased markedly from ~350 ± 100 to ~1200 ± 400 ppm across the T-OAE[10]. A comparable peak value (~1200 ppm) was recently reconstructed from the Haojiagou section in the Junggar Basin (China), using a similar stomatal proxy approach[11]. However, both records are characterized by stratigraphic gaps, sparse data coverage, and limited chronological control, which together preclude robust reconstruction of continuous pCO_2 evolution through the early Toarcian[10,11]. Substantially higher peak pCO_2 values (up to ~2400 ppm) have been proposed based on $\delta^{13}C$ measurements of pedogenic carbonates from the Shaping section, Sichuan Basin (China)[12]. However, these soil-based estimates are subject to large uncertainties arising from poorly constrained parameters in the inversion framework (e.g., soil CO_2 concentrations and depth-dependent $\delta^{13}C$ gradients), resulting in wide error bounds (1457–3315 ppm)[12]. These uncertainties are further exacerbated by a low temporal resolution (1 Myr) with a poor chronostratigraphic correlation between the local N-CIE and the well-established N-CIE in Europe[12].

On the basis of differences in N-CIE magnitudes recorded in terrestrial plants and marine substrates, a doubling of pCO_2 (from ~500 ppm to ~1000 ppm) was estimated across the T-OAE[5]. However, a high-resolution pCO_2 record spanning the entire event is still lacking, rendering quantitative constraints on carbon sources and release histories inherently uncertain[5,13]. Recently, Nordt et al.[14] reconstructed a higher-resolution and well-dated pCO_2 record over the early Toarcian stage using a C_3-plant CO_2 proxy from the Peniche section, Lusitanian Basin (Portugal). This new record provides a critical opportunity to better constrain the timing, magnitude, and tempo of pCO_2 change, thereby enabling improved quantitative reconstructions of carbon-cycle perturbations during the T-OAE.

Estimates of organic carbon burial during the T-OAE

Previous estimates of organic carbon (OC) burial during the T-OAE have been derived primarily from sedimentary total organic carbon (TOC) contents and redox-sensitive trace metal proxies. Based on the changes in TOC, it has been proposed that ~9000 Pg C of extra OC was buried in shallow marine environments during the T-OAE[15], including at least ~2600 Pg C within the European Epicontinental Shelf[16]. By contrast, reconstructions of the global ocean molybdenum (Mo) reservoir across the T-OAE, combined with compiled Mo/TOC records and their empirically derived modern relationship, yielded substantially higher estimates of global OC burial, reaching up to ~244,000 Pg C through the event[17].

Despite their utility, both approaches are subject to considerable uncertainties. TOC-based estimates rely predominantly on records from shallow basins in the European Tethyan realm, introducing a strong geographic bias that may distort the global OC burial signal[15]. Mo-based estimates are sensitive to assumptions of a temporally invariant global ocean Mo reservoir and Mo input fluxes within prescribed time intervals[17], assumptions that may not be valid under the markedly perturbed redox conditions of the T-OAE[15]. In addition, post-depositional thermal alteration can result in substantial loss of sedimentary organic matter[18], systematically modifying Mo/TOC-based reconstructions of OC burial through the event. Collectively, these methodological limitations highlight the need for an alternative, globally integrated approach that does not rely on local sedimentary proxies alone to quantify OC burial during the T-OAE.

2. Additional model outputs

Results from the maximum-pCO_2 (experiment 4) and alternative age-alignment tests (experiments 5–7) are presented in Supplementary Figs. 3 and 4 and summarized in Supplementary Table 2. Their implications for carbon-release magnitude, temporal structure and source composition are discussed in the main text.

The use of higher pCO_2 values to force the model is associated with greater rates and cumulative masses of carbon sequestration. The experiment using the smoothed upper pCO_2 reconstruction (experiment 3) produces greater OC-burial rates and masses than the simulations using the median and lower reconstructions (experiments 2 and 1, respectively; Supplementary Table 2). Weathering-driven excess carbon consumption

increases similarly with forcing magnitude (Fig. 3d), reflecting the stronger carbon-removal response required following greater carbon input.

Spatial distributions of modelled SST and seawater pH changes during the T-OAE using the smoothed lower and upper pCO_2 profiles are shown in Supplementary Fig. 5. Overall, both scenarios exhibit spatial patterns similar to those using the smoothed median pCO_2 profile (Fig. 5c and f), with differences primarily in amplitude reflecting the imposed carbon-release magnitude. In both experiments, modelled SST shows pronounced latitudinal amplification, with stronger warming at high latitudes than at low- and mid-latitudes (lower pCO_2 scenario: ~4–5°C at ~60°S versus ~2–3°C between 30°S and 30°N; upper pCO_2 scenario: ~6–7°C versus ~3–4°C) (Supplementary Fig. 5a and c). Modelled seawater pH likewise indicates global ocean acidification during the T-OAE, with larger pH declines at high latitudes (0.2–0.3 units using the smoothed lower pCO_2 profile and 0.25–0.35 units using the smoothed upper pCO_2 profile) compared to low- and mid-latitudes (≤0.2 units) (Supplementary Fig. 5b and d).

3. Geological evidence for temporally variable volatile release during Karoo–Ferrar magmatism

Geological observations from both the Karoo and Ferrar large igneous provinces provide evidence that volatile release may have occurred through multiple intrusive and degassing episodes rather than a single eruptive event. In the Karoo Basin, extensive sill complexes, hydrothermal vent structures, and sediment-hosted intrusion systems have been documented[19,20]. Thermal modelling further indicates that repeated and closely spaced sill emplacement could prolong and enhance contact metamorphism and volatile generation through successive heating of organic-rich sedimentary successions and coal-bearing strata[19,20]. Complementary studies of the Ferrar province document extensive intrusive plumbing systems and locally multiple shallow-level sill emplacement[21,22], indicating prolonged and temporally variable magmatic activity broadly synchronous with the T-OAE. Together, these observations suggest that carbon release associated with Karoo–Ferrar magmatism likely involved multiple intrusive and degassing episodes, providing geological support for temporally variable carbon release during the T-OAE.

Supplementary Figures

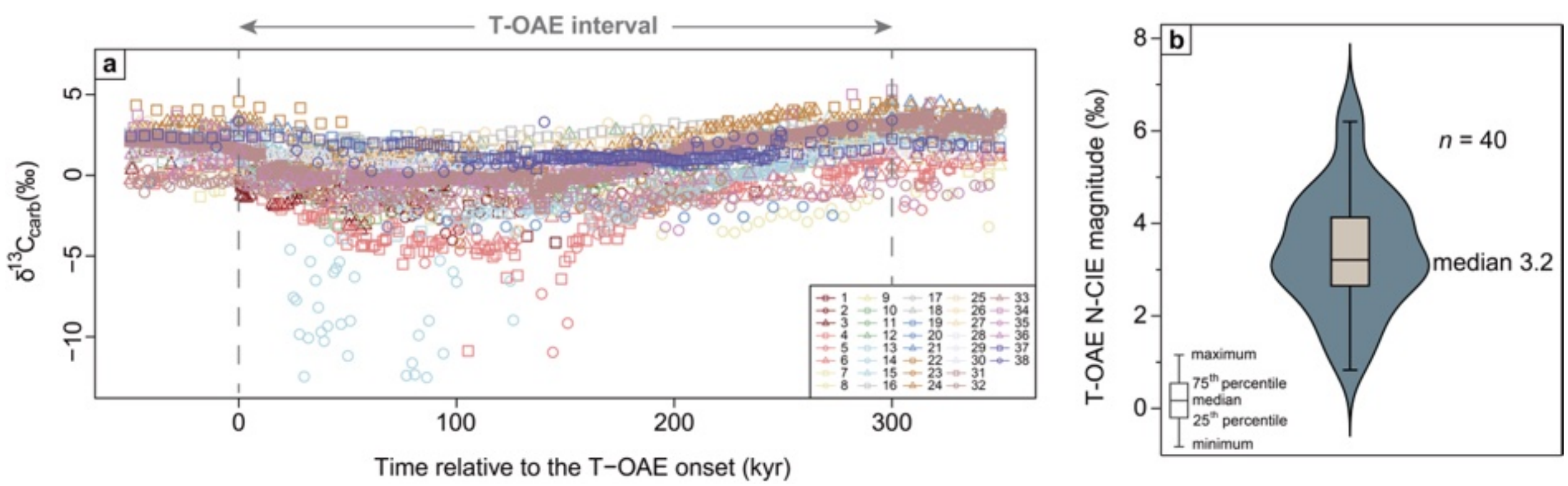

Supplementary Figure 1. Site-by-site compilation of $\delta^{13}C_{carb}$ records across the T-OAE. (a) $\delta^{13}C_{carb}$ records (*n* = 4048) from 38 profiles compiled in this study and projected onto the high-precision geochronology for the T-OAE[23]. References for the original $\delta^{13}C_{carb}$ records used are provided in Supplementary Table 1. Vertical grey dashed lines denote the onset and termination of the T-OAE. (b) Violin plot and boxplot showing the distribution of T-OAE N-CIE magnitudes derived from individual sections. The “*n*” value in panel b represents the number of compiled sections. Note that two additional sections (El Peñon and Asientos in Chile) are included because N-CIE magnitudes have been reported despite the incomplete stratigraphic records at these sites[24]. *T-OAE* Toarcian Oceanic Anoxic Event; *N-CIE* negative carbon isotope excursion.

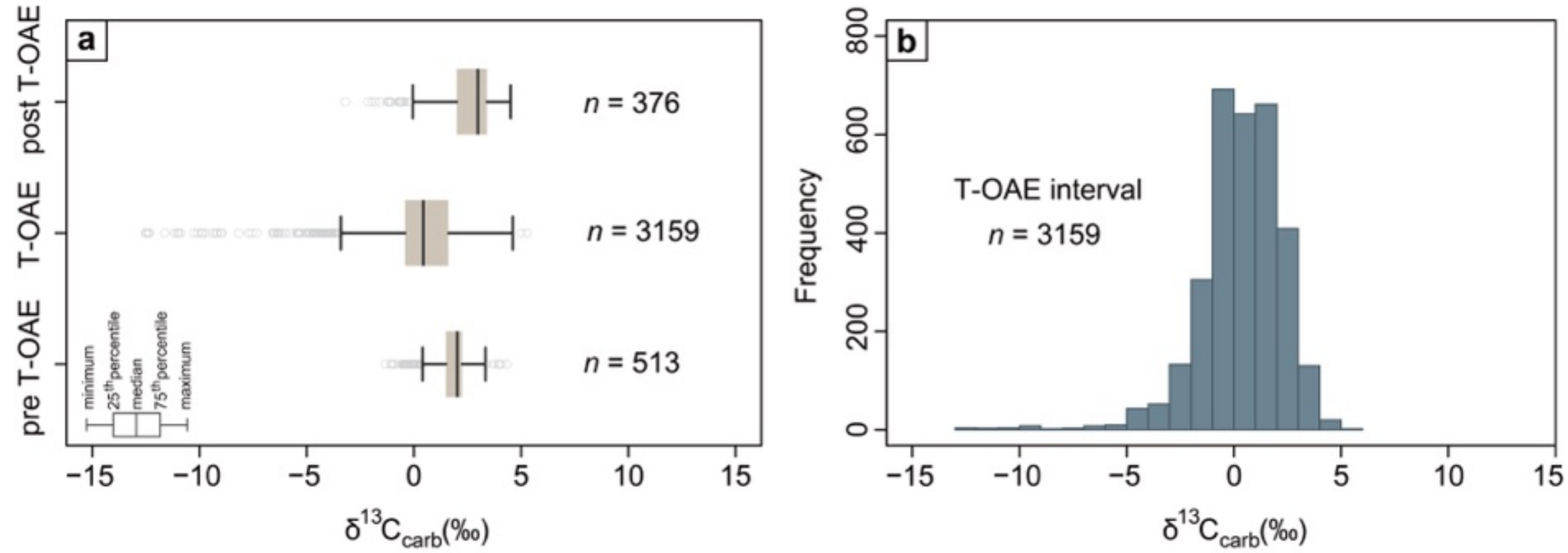

Supplementary Figure 2. Statistical analysis of compiled $\delta^{13}C_{carb}$ data across the T-OAE. (a) Boxplot illustrating the variability in compiled $\delta^{13}C_{carb}$ pre (−50 kyr to 0 kyr, median +2.02‰), during (0 kyr to 300 kyr, median +0.45‰), and post the T-OAE (300 kyr to 350 kyr, median +2.98‰). (b) Frequency distribution of $\delta^{13}C_{carb}$ during the T-OAE. The "*n*" value in panels a and b represents the number of $\delta^{13}C_{carb}$ data. *T-OAE* Toarcian Oceanic Anoxic Event.

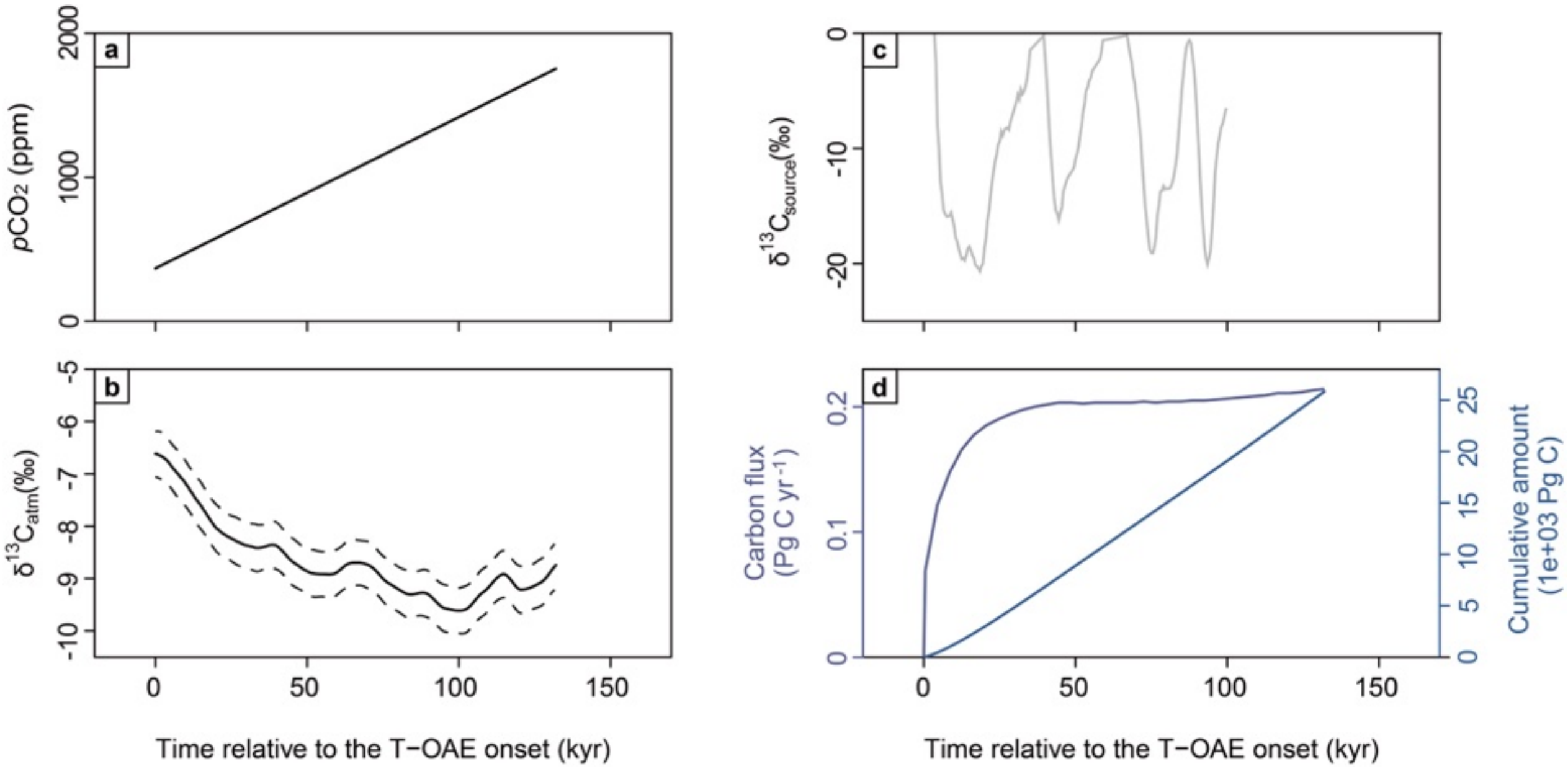

Supplementary Figure 3. Sensitivity experiment using the maximum proxy-derived *p*CO_2 forcing during the T-OAE. (a) Alternative *p*CO_2 forcing constructed using the maximum value (~1750 ppm) from the upper (84th percentile) reconstruction of Nordt et al.[14]. The trajectory to this maximum value from the corresponding *p*CO_2 value at the N-CIE onset (~370 ppm in the smoothed upper *p*CO_2 scenario at t = 0 kyr) was linearly interpolated onto the same age framework as the $\delta^{13}C_{atm}$ record. (b) Smoothed $\delta^{13}C_{atm}$ forcing used in the simulations (solid line). Dashed lines denote the 95% confidence interval. (c) Modelled temporal evolution of $\delta^{13}C_{source}$. (d) Modelled carbon release rates and cumulative masses. *T-OAE* Toarcian Oceanic Anoxic Event; *N-CIE* negative carbon isotope excursion. Numerical results are summarized in Supplementary Table 2.

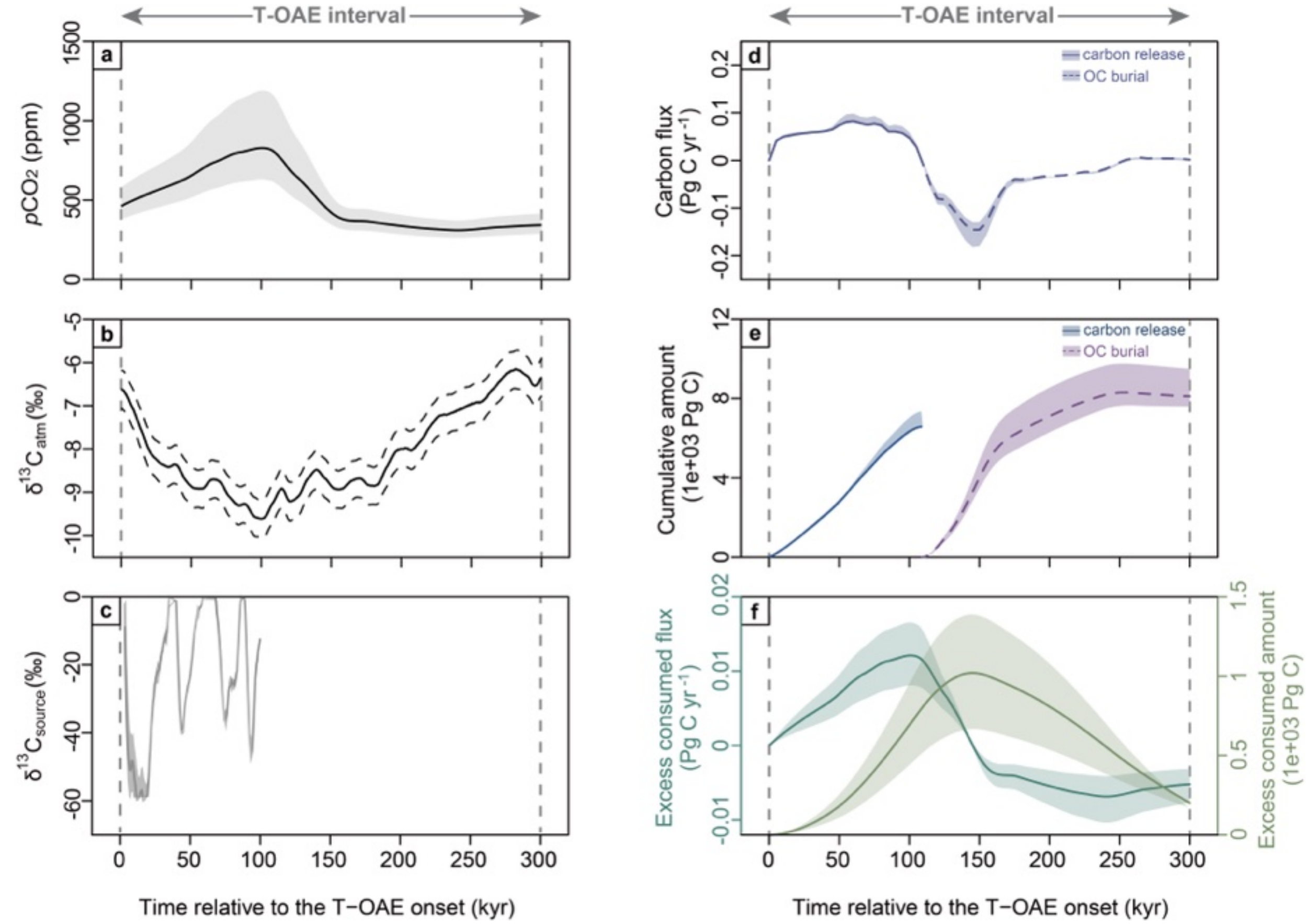

Supplementary Figure 4. Sensitivity experiments on age alignment between $p\mathrm{CO_2}$ and $\delta^{13}\mathrm{C_{atm}}$ during the T-OAE. (a) Smoothed $p\mathrm{CO_2}$ forcing used in the age-alignment experiment. The $p\mathrm{CO_2}$ maximum is shifted forward by 35 kyr to coincide with the minimum $\delta^{13}\mathrm{C_{atm}}$ at 100 kyr. Black solid line denotes the smoothed median $p\mathrm{CO_2}$ profile, and the shaded area denotes the smoothed lower (16th percentile) and upper (84th percentile) $p\mathrm{CO_2}$ profiles (see main text). (b) Smoothed $\delta^{13}\mathrm{C_{atm}}$ forcing used in the simulations. The minimum $\delta^{13}\mathrm{C_{atm}}$ remains fixed relative to the standard experiment. Black dashed lines denote the 95% confidence interval. (c) Modelled temporal evolution of $\delta^{13}\mathrm{C_{source}}$. (d) Modelled rates of carbon release and OC burial. (e) Modelled cumulative carbon-release masses and OC burial masses. (f) Modelled rates and masses of excess carbon consumption due to enhanced continental silicate weathering. Shaded envelopes in panels c–f represent the results using the smoothed lower and upper $p\mathrm{CO_2}$ profiles (experiments 5 and 7; Supplementary Table 2). *T-OAE* Toarcian Oceanic Anoxic Event; *OC* organic carbon.

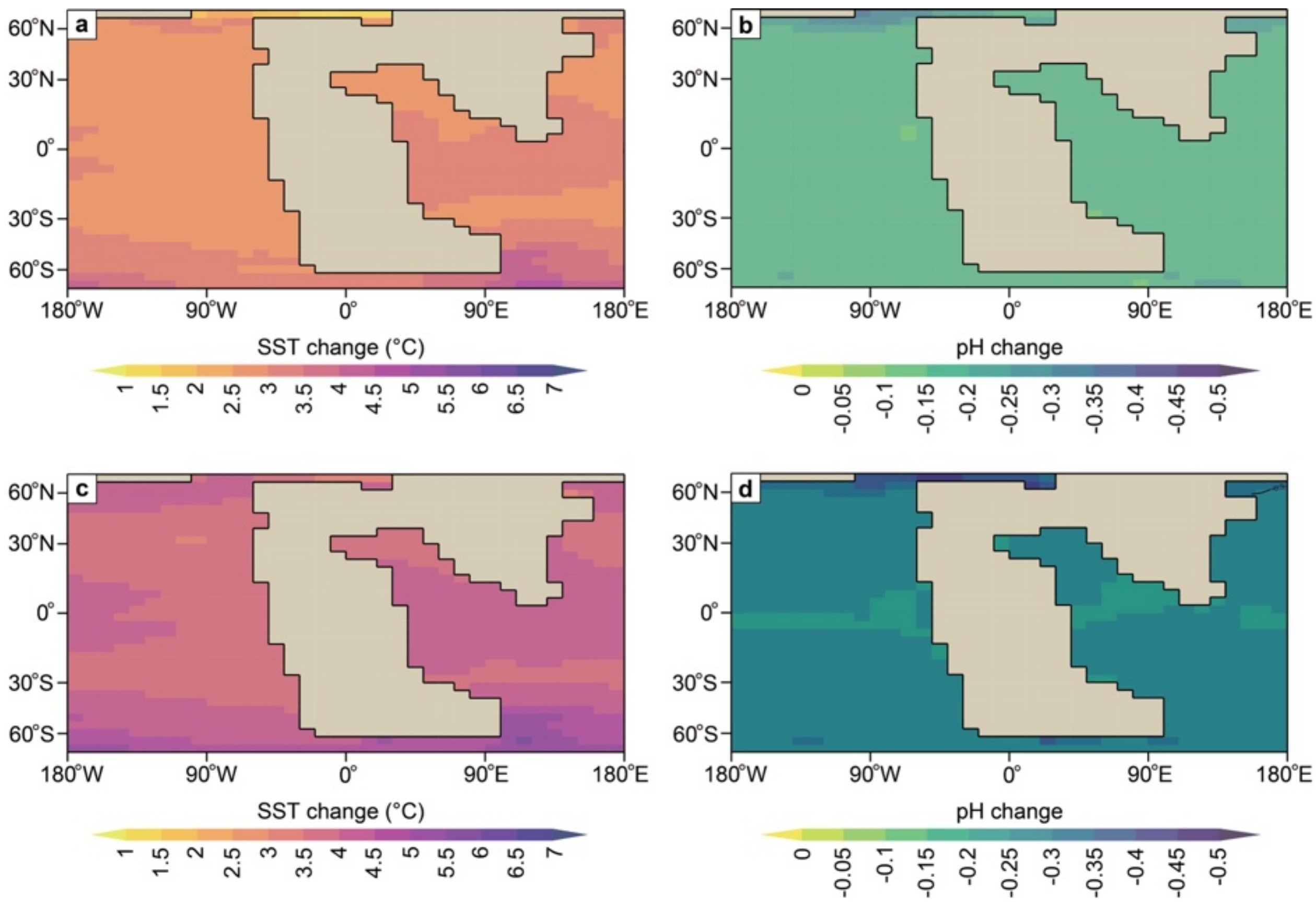

Supplementary Figure 5. Modelled spatial patterns of SST and seawater pH changes across the T-OAE. (a) Modelled global SST changes using the smoothed lower pCO_2 profile (experiment 1). (b) Modelled global seawater pH changes using the smoothed lower pCO_2 profile (experiment 1). (c) Modelled global SST changes using the smoothed upper pCO_2 profile (experiment 3). (d) Modelled global seawater pH changes using the smoothed upper pCO_2 profile (experiment 3). *T-OAE* Toarcian Oceanic Anoxic Event; *SST* sea surface temperature.

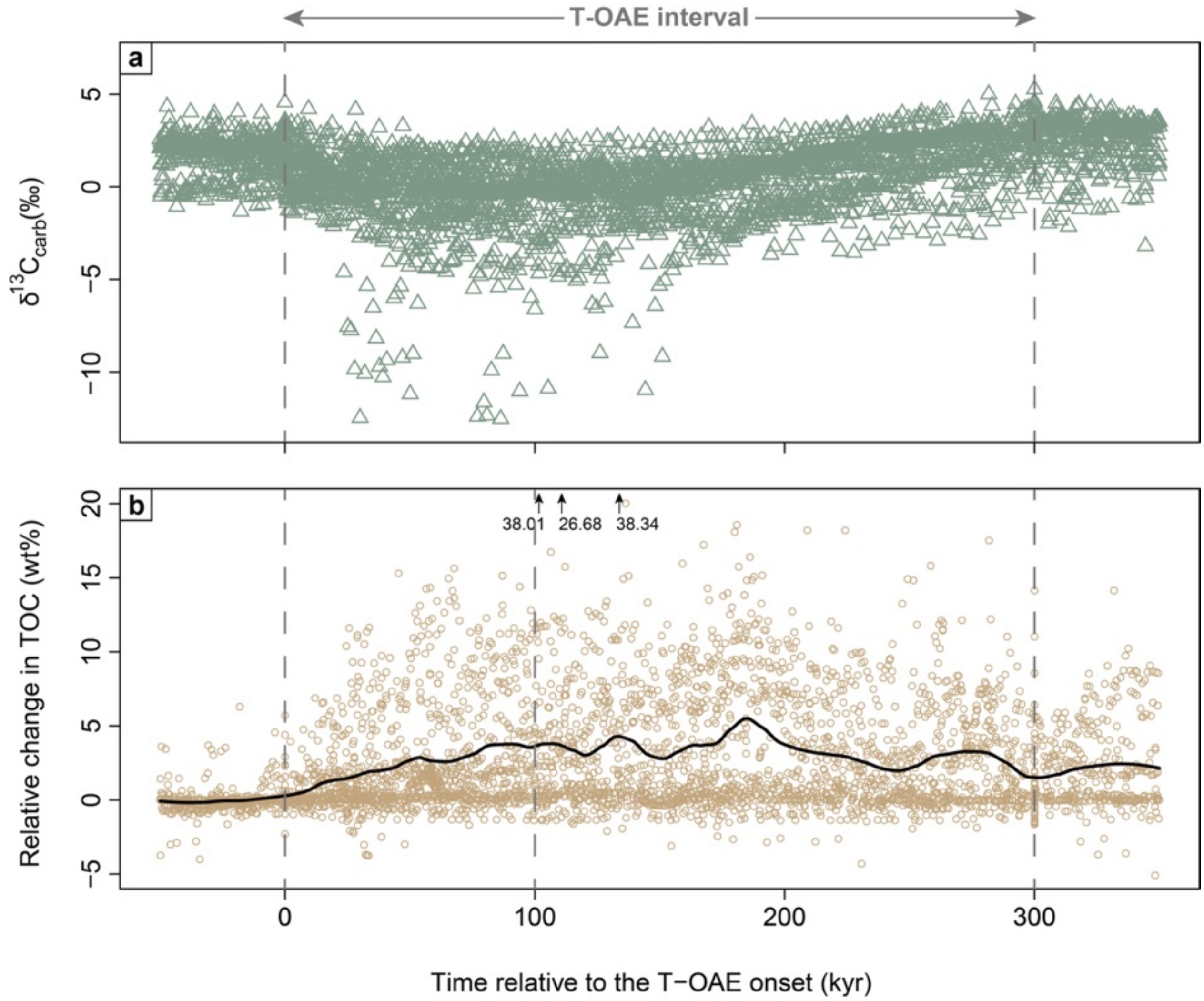

Supplementary Figure 6. Compiled geochemical data across the T-OAE. (a) Compilation of $\delta^{13}C_{carb}$ records (n = 4048) from 38 profiles projected onto the age model adopted in this study. (b) Relative TOC enrichment (n = 3832) compiled from 64 globally distributed marine sections based on the dataset of Kemp et al.[15]. Relative TOC enrichment was calculated by subtracting the mean pre-event TOC value (−50 to 0 kyr relative to the onset of the T-OAE) from individual TOC measurements within each section. The solid line represents a LOESS fit (span = 0.1) to the stacked dataset, which tracks first-order changes in global TOC enrichment across the T-OAE. The vertical grey dashed line at 100 kyr separates the onset (0–100 kyr) and recovery (100–300 kyr) intervals. *T-OAE* Toarcian Oceanic Anoxic Event; *TOC* total organic carbon.

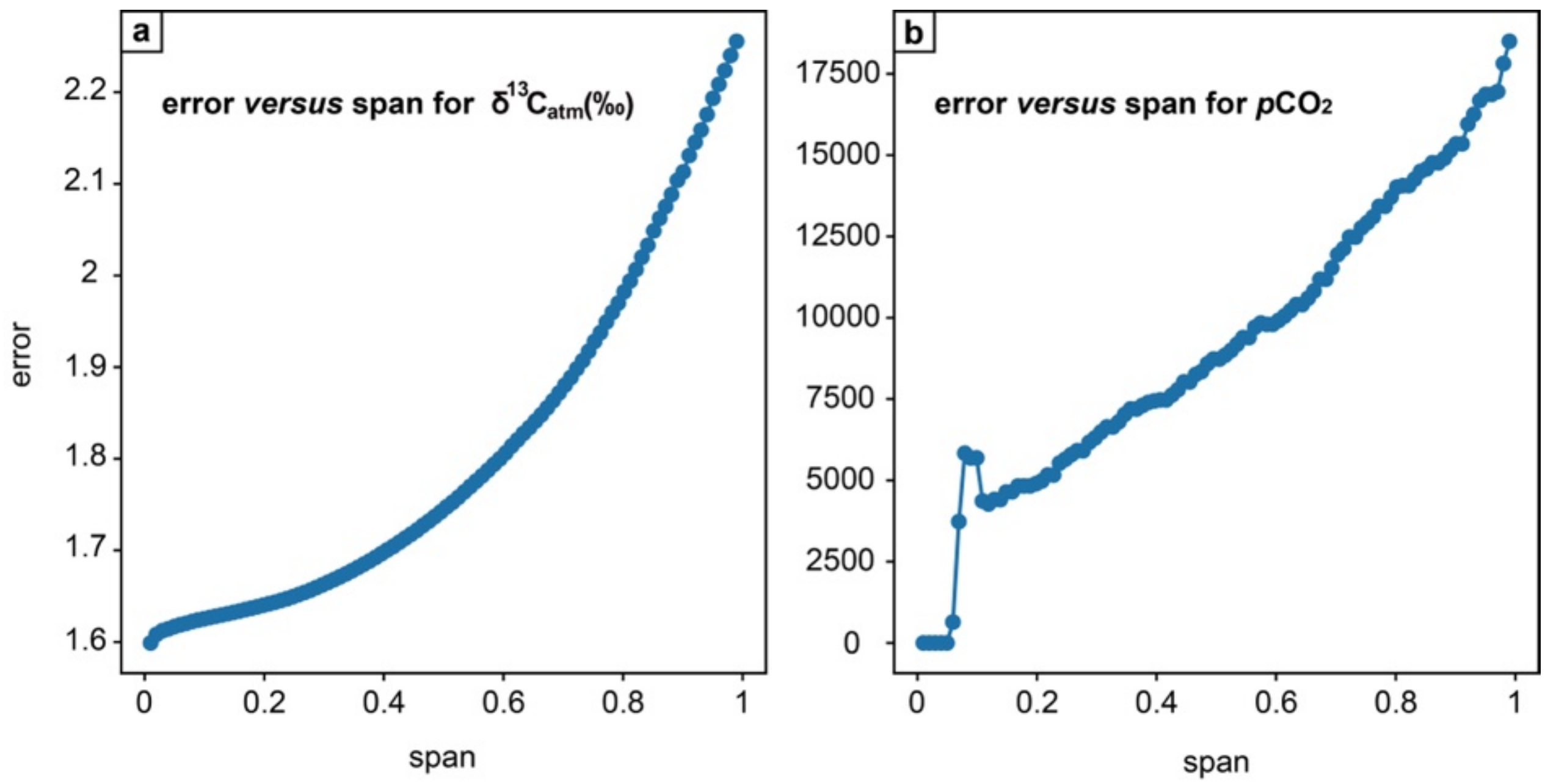

Supplementary Figure 7. Span sensitivity analyses of the LOESS fit method. (a) The analysis of the model input variable $\delta^{13}C_{atm}$. (b) The analysis of the model input variable pCO_2. The fitting error (y-axis) is quantified as the mean squared error between the LOESS-smoothed curve and the original dataset across a range of span values (0.01–0.99).

Supplementary Tables

Supplementary Table 1. List of study sites, including site names, numerical identifiers used in Fig. 1a, and the corresponding thickness and magnitude of the T-OAE N-CIE recorded in inorganic carbon reservoirs.

Site number	Site name	N-CIE thickness (m)	N-CIE magnitude (‰)	Reference
1	Mochras	33.05	4.0	Jenkyns et al. (2001)
2	Sancerre	4.83	4.9	Hermoso et al. (2012)
3	Lorraine	3.37	4.8	Ruebsam et al. (2014)
4	Denkingen	4.06	6.2	Suan et al. (2015)
5	Dotternhausen	3.52	5.7	Röhl et al. (2001)
6	Rietheim	1.21	3.4	Montero-Serrano et al. (2015)
7	Riniken	1.70	3.0	Fantasia et al. (2019)
8	Gipf	1.08	4.1	Fantasia et al. (2019)
9	Creux de l'Ours	3.94	2.3	Fantasia et al. (2019)
10	Cuers	6.34	4.0	Leonide et al. (2012)
11	Dogna	17.10	4.1	Jenkyns et al. (2001)
12	Breggia	1.26	1.6	Fantasia et al. (2019)
13	Sogno	10	2.9	Erba et al. (2022)
14	Gajum	19.42	4.4	Erba et al. (2022)
15	Colma di Malcesine	31	1.3	Woodfine et al. (2008)
16	Sega d'Ala	36	2.0	Woodfine et al. (2008)
17	Valdorbia	23.80	4.5	Sabatino et al. (2009)
18	Gornje Jelenje	35.30	3.1	Sabatino et al. (2013)
19	Plitvice Spring	18.90	3.1	Brčić et al. (2024)
20	Bjelopolie	66	4.8	Brčić et al. (2024)
21	Velebit-A	34.80	3.3	Sabatino et al. (2013)
22	Velebit-B	31.80	4.3	Sabatino et al. (2013)
23	Kastelli	6.50	6.5	Kafousia et al. (2011)
24	Toka	11.90	3.9	Kafousia et al. (2014)
25	Petousi	6.50	0.8	Kafousia et al. (2014)
26	Mercato San Severino	12.20	2.4	Trecalli et al. (2012)
27	Monte Sorgenza	9.80	2.9	Trecalli et al. (2012)
28	Es Cosconar 4	7.30	1.8	Rosales et al. (2018)
29	La Cerradura	9.35	1.2	Ruebsam et al. (2020)
30	Rabacal	13.30	3.3	Pittet et al. (2014)
31	Peniche	20.16	3.6	Hesselbo et al. (2007)
32	Talghemt	16.05	2.4	Boulila et al. (2019)
33	Amellago	50	2.7	Bodin et al. (2010)
34	Toksine	82.52	4.6	Bodin et al. (2016)
35	Bilong Co	14.70	2.9	Fu et al. (2016)
36	Suobucha	55	3.1	Nie et al. (2023)
37	Qixiangcuo	48.50	2.0	Ji et al. (2025)
38	Well AR	23.10	3.2	De Keyser et al. (2025)

Supplementary Table 2. Summary of cGENIE model experiments conducted in this study. Experiments 1–3 use the smoothed lower, median, and upper pCO_2 reconstructions of Nordt et al.[14]. Experiment 4 tests the effect of potential attenuation of peak pCO_2 caused by LOESS smoothing by employing an alternative trajectory constrained by the maximum proxy-derived pCO_2 value (~1750 ppm) (see also Supplementary Fig. 3). Experiments 5–7 evaluate uncertainties associated with the temporal alignment between pCO_2 and $\delta^{13}C_{atm}$ (see also Supplementary Fig. 4).

Experiment ID	*p*CO2 forcing	age alignment	mean $\delta^{13}C_{source}$ (‰)	minimum $\delta^{13}C_{source}$ (‰)	cumulative release mass (Pg C)	peak release flux (Pg C yr^{-1})	average release flux (Pg C yr^{-1})	peak OC burial flux (Pg C yr^{-1})	OC burial mass (Pg C)
1	lower	no shift	−12.6	−32.1	18,802	0.16	0.12	0.11	5701
2	median	no shift	−13.6	−33.0	17,849	0.16	0.12	0.14	6838
3	upper	no shift	−13.7	−34.7	18,756	0.18	0.13	0.19	9000
4	maximum	no shift	−10.7	−20.4	25,824	0.21	N/A	N/A	N/A
5	lower	a 35 kyr forward shift of *p*CO2	−24.9	−55.0	6586	0.08	0.06	0.13	7649
6	median	a 35 kyr forward shift of *p*CO2	−25.1	−59.9	6569	0.08	0.06	0.15	8300
7	upper	a 35 kyr forward shift of *p*CO2	−21.7	−59.5	7342	0.10	0.07	0.18	9759

N/A, Not Available

Supplementary References

1. Suan, G., van de Schootbrugge, B., Adatte, T., Fiebig, J. & Oschmann, W. Calibrating the magnitude of the Toarcian carbon cycle perturbation. *Paleoceanography* **30,** 495–509 (2015).
2. Hesselbo, S. P. et al. Massive dissociation of gas hydrate during a Jurassic oceanic anoxic event. *Nature* **406,** 392–395 (2000).
3. Kemp, D. B., Coe, A. L., Cohen, A. S. & Schwark, L. Astronomical pacing of methane release in the Early Jurassic period. *Nature* **437**, 396–399 (2005).
4. Xu, W. et al. Carbon sequestration in an expanded lake system during the Toarcian oceanic anoxic event. *Nat. Geosci.* **10,** 129–134 (2017).
5. Ruebsam, W., Reolid, M. & Schwark, L. $\delta^{13}C$ of terrestrial vegetation records Toarcian CO_2 and climate gradients. *Sci. Rep.* **10,** 117 (2020).
6. French, K. L., Sepúlveda, J., Trabucho-Alexandre, J., Gröcke, D. R. & Summons, R. E. Organic geochemistry of the early Toarcian oceanic anoxic event in Hawsker Bottoms, Yorkshire, England. *Earth Planet. Sci. Lett.* **390,** 116–127 (2014).
7. Jin, X. et al. The Jenkyns Event (early Toarcian OAE) in the Ordos Basin, North China. *Glob. Planet. Change* **193,** 103273 (2020).
8. Wang, Y. et al. Toarcian greenhouse warming shifted climate belts poleward with global change implications. *J. Geophys. Res. Atmos.* **130,** e2024JD043219 (2025).
9. Wu, Y. et al. Microbial metabolism amplified warming in three Phanerozoic hyperthermal events. *Nat. Commun.* **16,** 11372 (2025).
10. McElwain, J. C., Wade-Murphy, J. & Hesselbo, S. P. Changes in carbon dioxide during an oceanic anoxic event linked to intrusion into Gondwana coals. *Nature* **435,** 479–482 (2005).
11. Li, T., Yang, X. & Deng, S. Palaeo-CO_2 estimation of the Early Jurassic based on Cheirolepidiaceous conifer in the Junggar Basin, Xinjiang, China. *Acta Palaeontol. Sin.* **60,** 253–262 (2021).
12. Li, X. et al. Early Jurassic climate and atmospheric CO_2 concentration in the Sichuan paleobasin, southwestern China. *Clim. Past* **16,** 2055–2074 (2020).
13. Beerling, D. J. & Brentnall, S. J. Numerical evaluation of mechanisms driving Early Jurassic changes in global carbon cycling. *Geology* **35,** 247–250 (2007).
14. Nordt, L., Breecker, D. & White, J. Jurassic greenhouse ice-sheet fluctuations sensitive to atmospheric CO_2 dynamics. *Nat. Geosci.* **15,** 54–59 (2022).
15. Kemp, D. B., Suan, G., Fantasia, A., Jin, S. & Chen, W. Global organic carbon burial during the Toarcian oceanic anoxic event: Patterns and controls. *Earth-Sci. Rev.* **231,** 104086 (2022).
16. Ruvalcaba Baroni, I. et al. Ocean circulation in the Toarcian (Early Jurassic): a key control on deoxygenation and carbon burial on the European Shelf. *Paleoceanogr. Paleoclimatol.* **33,** 994–1012 (2018).
17. Them, T. R. et al. Reduced marine molybdenum inventory related to enhanced organic carbon burial and an expansion of reducing environments in the Toarcian (Early Jurassic) oceans. *AGU Advances* **3,** e2022AV000671 (2022).

18. Dickson, A. J., Idiz, E., Porcelli, D. & van den Boorn, S. H. J. M. The influence of thermal maturity on the stable isotope compositions and concentrations of molybdenum, zinc and cadmium in organic-rich marine mudrocks. *Geochim. Cosmochim. Acta* **287,** 205–220 (2020).
19. Svensen, H., Jamtveit, B., Planke, S. & Chevallier, L. Structure and evolution of hydrothermal vent complexes in the Karoo Basin, South Africa. *J. Geol. Soc.* **163,** 671–682 (2006).
20. Svensen, H. et al. Hydrothermal venting of greenhouse gases triggering Early Jurassic global warming. *Earth Planet. Sci. Lett.* **256,** 554–566 (2007).
21. Burgess, S. D., Bowring, S. A., Fleming, T. H. & Elliot, D. H. High-precision geochronology links the Ferrar large igneous province with early-Jurassic ocean anoxia and biotic crisis. *Earth Planet. Sci. Lett.* **415,** 90–99 (2015).
22. Elliot, D. H. & Fleming, T. H. Physical volcanology and geological relationships of the Jurassic Ferrar large igneous province, Antarctica. *J. Volcanol. Geotherm. Res.* **172,** 20–37 (2008).
23. Kemp, D. B. et al. The timing and duration of large-scale carbon release in the Early Jurassic. *Geology* **52,** 891–895 (2024).
24. Fantasia, A. et al. The Toarcian oceanic anoxic event in southwestern Gondwana: an example from the Andean Basin, northern Chile. *J. Geol. Soc. Lond.* **175,** 883–902 (2018).
