# Supplementary material for: Massive release of predominantly magmatic carbon drove an Early Jurassic hyperthermal
Source: Nat Commun. 2026 Sep 18;17:10968. doi: 10.1038/s41467-026-77747-y (PMC13683373; doi:10.1038/s41467-026-77747-y)
Supplement: Supplementary file 2 [file 41467_2026_77747_MOESM2_ESM.pdf]

nature portfolio

Peer Review File

Massive release of predominantly magmatic carbon drove an Early Jurassic hyperthermal

Corresponding Author: Professor David Kemp

This file contains all reviewer reports in order by version, followed by all author rebuttals in order by version.

Version 0:

Reviewer comments:

Reviewer #1

(Remarks to the Author)
Chen et al present new modelling results and data compilation for the Toarcian Oceanic Anoxic Event interval in the early Jurassic. By using a dual inversion for both d13C and pCO2 proxy data, their cGENIE model results constrain both the amount and the isotopic composition of carbon input (and burial) required to drive the observed negative carbon isotope excursion. The manuscript is well-written and easy to understand (which is a strong positive!).

I think this approach is valuable and this study potentially important. However, the study needs to be more clear about some of their assumptions and more rigorous in their uncertainty estimates. My primary concern is uncertainty propagation and reporting.

Bringing in additional quantitative reasoning will strengthen the study and confidence in their modelling results, and it will also significantly increase the novelty of their findings. At the moment, I think this study is interesting, but it is not significantly advancing our understanding of the event, given that several other modelling studies have also tried to reproduce the T-OAE. To that end, more explicit discussion of other modeled scenarios is needed. For example, the GEOCLIM results already mentioned (Heimdal et al., 2021), which, although a forward modeling approach rather than an inversion, also explicitly incorporated both d13C and pCO2 proxy records (as this study does). How does cGENIE differ from GEOCLIM? How do their constraints and results agree/disagree? E.g., L256/257 is great but why are different pCO2 values used between the two studies? Similar comparisons with the other mentioned modeling results would also be helpful.

Uncertainty propagation:
-I am confused as to how the uncertainty in the model results (Fig 3D and E) for C emissions is so small compared to the uncertainty in the pCO2 data – which seems as though it spans ~500 - >1000 ppm during the OAE. I understand from L107 that three different pCO2 scenarios were used to create this uncertainty. Does this correspond with the span of pCO2 values? The range of uncertainty in the modeled C emissions seems too small relative to the variability seen in pCO2 records.
-The variability in carbon isotope data seen in Fig 2B is significant. It is not clear to me how this variability is taken into account in the modeling results (or even in the calculated d13C atm values in Fig 3A). Assuming that spatial variability (between sites in the compilation) is a primary driver of the scatter in the overall $\delta^{13}C$ data, it would be better to analyze the magnitude of the shift (excursion) at each specific location rather than calculating a global average of d13C at each time point (I think that this is what is implied by Fig 2C/D). This site-by-site approach would also reduce errors due to age-model uncertainties, since plotting all records on a single timeline falsely assumes their dates align perfectly (which is unlikely given this study's assumption of linear age progression between CIE initiation and recovery across all sites).
-L 134 The calculation of d13C atm from the carbonate records should have some uncertainty that can be propagated forward.

My secondary concern is the lack of agreement between model results and the stratigraphic record. Is this carbon flux (both emissions and burial) at all reasonable given what we know about the Karoo/Ferrar, magma/thermogenic volatile release, and carbon burial associated with the OAE?
L330: -Is it then necessary to invoke an increase in OC burial earlier in the CIE (and hence larger C emissions to balance the effects of this burial)? I suspect that this could be prescribed, or at least the effects of this calculated (again this should be incorporated, at least descriptively, into the uncertainty reported for the model results – including in the figures. Right now,

the carbon emissions/output seems invalidated by this observation. It is not sufficient, in my opinion, to say that the modelled flux is a "lower bound". Would this increase the proportion (or decrease the isotopic composition) of the necessary emissions? I think that a rigorous discussion of this is required in order for the model results to be useful – and indeed to demonstrate that majority magmatic carbon is sufficient (as claimed in the title).
-The negative CIE associated with the T-OAE is superimposed on a broader positive excursion (or at least shift – see e.g., Storm et al., 2020) – I find this hard to reconcile with the model results, in particular that increased organic burial is only invoked starting ~150 kyr after the n-CIE onset. Even if it is beyond scope to model this longer time interval, the timing of increased organic burial should be explicitly discussed (as noted in the previous comment).
-The Karoo/Ferrar LIP and thermogenic carbon release are discussed (e.g. L190), but these discussions are fairly abstract. Is it possible to use any of what we know about the volcanism or thermogenic volatile release to constrain or evaluate the model results in a quantitative way?

(Remarks on code availability)

Reviewer #2

(Remarks to the Author)
Review of "massive release of predominantly magmatic carbon drove an Early Jurassic hyperthermal" by Chen et al., submitted to Nature Communications

I enjoyed reading the manuscript by Chen and co-authors. The manuscript is well written, easy to follow, and the authors clearly communicate the methods used, and the motivation behind the study. The study is robust and novel and provides constraints on carbon cycle dynamics during the T-OAE using the cGENIE model. Importantly, the authors take uncertainties of proxy records and carbon release (e.g., timing, isotopic signature, C mass/flux etc.) into account by providing sensitivity analyses, which is key in modeling studies. They also discuss simplifications, potential shortcomings and limitations in their study. Efforts in improving our understanding of past events of climatic and environmental disruptions, and the impacts from carbon sources, are important. The last section (implications for modern global warming) nicely ties their results, and the importance of understanding past climate/biotic disruptions, to the challenges we are facing in the present. I would be happy to recommend this manuscript for publication with minor changes. See comments below:

Line 88: Please specify that references for the isotope records can be found in Supplementary Table 1. Please also add this in the legend of Figure 1 and 2.

Line 272: Could you please also add the experiment ID so that it is easy to compare to Supplementary Table 2. This comment applies not only here, but throughout the manuscript.

Line 875: I recommend to separate panel A-B from C-F; I find it a bit confusing to show both model inputs and outputs in the same figure. I believe there are constraints on the number of figures allowed. Since Fig. 2 already shows model inputs; you could add the d13C curve from Fig. 3A to Fig. 2B, and the pCO2 curve in Fig. 3B to Fig 2A. Give the curves + uncertainty range a different color, dashed line etc. In addition to separating inputs from outputs + reducing the number of figure panels, this would also provide direct comparisons between the proxy data and the smoothed curves that you feed the model with.

Line 886: It is a bit difficult to see the temperature increases due to the large range in values. Removing all white space would be helpful – for example you can start your y-axis at 10 degrees C.

Line 320: I would recommend not to abbreviate the end-Permian mass extinction.

(Remarks on code availability)
When I click the link I am not given access, so I have not been able to review the code.

Version 1:

Reviewer comments:

Reviewer #1

(Remarks to the Author)
The authors have thoughtfully responded to my critique and have made a significant effort to address it, including the addition of the TOC compilation, additional uncertainty estimates and propagation, and comparison with other published models and TOAE carbon emissions.
I have a few outstanding comments.
"These results help to underline that uncertainties associated with reconstructed pCO2 values during the T-OAE exert only a limited influence on modelled carbon-release histories and source characteristics"
This makes it seem like the required model forcing you need is not very sensitive to the pco2 constraint. Is that the case?
L405: Consequently, although OC burial during the N-CIE onset phase is not captured by our inversion approach, available stratigraphic records suggest that it likely constituted a smaller component of total event-scale OC burial than that associated with the recovery interval, and therefore is unlikely to substantially affect our model results.

Is it possible to do some kind of quantitative analysis, so it can be better than “unlikely”? How affected might it be?

(Remarks on code availability)

Open Access This Peer Review File is licensed under a Creative Commons Attribution 4.0 International License, which permits use, sharing, adaptation, distribution and reproduction in any medium or format, as long as you give appropriate credit to the original author(s) and the source, provide a link to the Creative Commons license, and indicate if changes were made.
In cases where reviewers are anonymous, credit should be given to 'Anonymous Referee' and the source.
The images or other third party material in this Peer Review File are included in the article’s Creative Commons license, unless indicated otherwise in a credit line to the material. If material is not included in the article’s Creative Commons license and your intended use is not permitted by statutory regulation or exceeds the permitted use, you will need to obtain permission directly from the copyright holder.
To view a copy of this license, visit https://creativecommons.org/licenses/by/4.0/

Response letter for NCOMMS-26-016106A: Massive release of predominantly magmatic carbon drove an Early Jurassic hyperthermal

We thank the editor and two reviewers for their constructive and insightful comments on our manuscript. Following their suggestions, we have carefully addressed all comments and queries raised (shown in black text) and provide detailed, point-by-point responses below (in blue text).

Reviewer #1 (Remarks to the Author):

Chen et al present new modelling results and data compilation for the Toarcian Oceanic Anoxic Event interval in the early Jurassic. By using a dual inversion for both d13C and pCO2 proxy data, their cGENIE model results constrain both the amount and the isotopic composition of carbon input (and burial) required to drive the observed negative carbon isotope excursion. The manuscript is well-written and easy to understand (which is a strong positive!).

I think this approach is valuable and this study potentially important. However, the study needs to be more clear about some of their assumptions and more rigorous in their uncertainty estimates. My primary concern is uncertainty propagation and reporting.

Bringing in additional quantitative reasoning will strengthen the study and confidence in their modelling results, and it will also significantly increase the novelty of their findings. At the moment, I think this study is interesting, but it is not significantly advancing our understanding of the event, given that several other modelling studies have also tried to reproduce the T-OAE. To that end, more explicit discussion of other modeled scenarios is needed. For example, the GEOCLIM results already mentioned (Heimdal et al., 2021), which, although a forward modeling approach rather than an inversion, also explicitly incorporated both d13C and pCO2 proxy records (as this study does). How does cGENIE differ from GEOCLIM? How do their constraints and results agree/disagree? E.g., L256/257 is great but why are different pCO2 values used between the two studies? Similar comparisons with the other mentioned modeling results would also be helpful.

We thank the reviewer for this important and constructive comment. We agree that a clearer and more quantitative comparison with previous modelling studies is essential to demonstrate the novelty and robustness of our approach.

Most previous studies employed forward modelling approaches (e.g., Beerling and Brentnall, 2007, *Geology*; Ullmann et al., 2020, *Scientific Reports*; Heimdal et al., 2021, *Nature Communications*), in which the magnitude, timing, and isotopic composition of carbon inputs are prescribed *a priori*. By contrast, our study is the first to apply a double-inversion framework that simultaneously assimilates $\delta^{13}C_{atm}$ and pCO_2 proxy records to directly reconstruct **the temporal evolution** of both carbon fluxes and their isotopic composition. This allows us to resolve **time-dependent**

variations in carbon sources, including transient or pulsed releases, without prescribing their structure in advance.

In addition, our use of the cGENIE Earth system model enables us to explore not only global carbon-cycle dynamics but also their **spatial expression** in the ocean–atmosphere system. The model's three-dimensional ocean structure allows us to examine **latitudinal and vertical gradients** in temperature and ocean chemistry (e.g., SST, pH, etc.), which are not well resolved in previous models. These spatial patterns provide additional, testable constraints on the mechanisms driving the T-OAE and highlight directions for future data-model integration.

As suggested, we have expanded the comparison with previous modelling studies in the revised manuscript. Specifically, we have added a new figure (Fig. 4) that allows direct comparison of previously published estimates of carbon release during the T-OAE alongside our cGENIE-based results. This provides a clearer overview of the range of inferred carbon-cycle perturbations across studies. We have also added more text discussing the differences between different modelling work. For example, although Heimdal et al. (2021) also proposed pulsed carbon release scenarios, these were implemented using prescribed forcing structures and evaluated against relatively sparse proxy datasets with substantial stratigraphic and chronological uncertainties (e.g., stomatal index-based pCO_2 reconstruction; McElwain et al., 2005, *Nature*). By contrast, our study benefits from a more recent and substantially improved pCO_2 reconstruction (Nordt et al., 2022, *Nature Geoscience*), and new high-precision geochronology (Kemp et al., 2024, *Geology*). Together, our work provides tighter constraints for model-data comparison and enables a more robust reconstruction of carbon-cycle dynamics.

Differences in model forcings likely contribute to the divergent estimates of carbon release among studies, particularly with respect to the reconstructed pCO_2 trajectories and N-CIE morphology. As suggested, we have now explicitly discussed these differences in the revised manuscript, including the reasons why different baseline/peak pCO_2 values and N-CIE morphology are used, and how these propagate into model outcomes. Taken together, these additions strengthen the quantitative framework of our study and highlight how our inversion-based approach improves upon previous modelling efforts by constraining the magnitude and timing of carbon-cycle perturbations, as well as the spatial patterns of their associated climatic and environmental responses during the T-OAE.

Uncertainty propagation:
-I am confused as to how the uncertainty in the model results (Fig 3D and E) for C emissions is so small compared to the uncertainty in the pCO2 data – which seems as though it spans ~500 - >1000 ppm during the OAE. I understand from L107 that three different pCO2 scenarios were used to create this uncertainty. Does this correspond with the span of pCO2 values? The range of uncertainty in the modeled

C emissions seems too small relative to the variability seen in pCO2 records.
We thank the reviewer for this insightful comment. To more quantitatively assess the sensitivity of our results to uncertainty in the pCO_2 reconstruction, we performed an additional sensitivity experiment (experiment 4) using an alternative pCO_2 trajectory based on the upper envelope of the proxy-derived pCO_2 reconstruction from Nordt et al. (2022), which reaches a maximum of ~1,750 ppm during the T-OAE. This scenario was implemented using simple linear interpolation and coupled with the same fitted $\delta^{13}C_{atm}$ forcing applied in the standard simulations (Supplementary Fig. 3), thereby retaining the full amplitude of reconstructed pCO_2 variability while maintaining temporal consistency between the two forcing records. Relative to the standard experiment (i.e., using the smoothed median pCO_2 profile as inputs; experiment 2), this maximum pCO_2 scenario yields somewhat higher carbon-release rates (~0.20 Pg C yr^{-1}) and cumulative carbon-release masses (~26,000 Pg C) as expected (Supplementary Table 2), but the overall magnitude of the inferred carbon-cycle perturbation remains broadly comparable. Likewise, the reconstructed carbon source (experiment 4) exhibits a similar average isotopic composition ($\delta^{13}C_{source}$ = ~−11‰; Supplementary Table 2) and retains the same pulsed-emission structure identified in the standard simulation. These results help to underline that uncertainties associated with reconstructed pCO_2 values during the T-OAE exert only a limited influence on modelled carbon-release histories and source characteristics. The principal conclusions of the study – including the requirement for substantial carbon release and the dominant contribution of relatively ^{13}C-enriched carbon sources consistent with a major magmatic component – hence remain robust across the tested pCO_2 scenarios. We have now clarified this in the revised manuscript.

-The variability in carbon isotope data seen in Fig 2B is significant. It is not clear to me how this variability is taken into account in the modeling results (or even in the calculated d13C atm values in Fig 3A). Assuming that spatial variability (between sites in the compilation) is a primary driver of the scatter in the overall $\delta^{13}C$ data, it would be better to analyze the magnitude of the shift (excursion) at each specific location rather than calculating a global average of d13C at each time point (I think that this is what is implied by Fig 2C/D). This site-by-site approach would also reduce errors due to age-model uncertainties, since plotting all records on a single timeline falsely assumes their dates align perfectly (which is unlikely given this study's assumption of linear age progression between CIE initiation and recovery across all sites).
We thank the reviewer for raising this important point. We agree that individual sections may exhibit significant inter-site variability, partly reflecting local depositional conditions, diagenetic overprinting, and thermal metamorphism. To clarify this point, we have now added a new Figure (Supplementary Fig. 1A) showing the $\delta^{13}C_{carb}$ evolution of each individual section included in the compilation. These comparisons demonstrate that the most anomalously negative $\delta^{13}C_{carb}$ values are largely restricted to a small number of sections (n = 3) previously recognized as strongly influenced by regional alteration processes (e.g., Röhl et al., 2001, *Palaeogeography*

Palaeoclimatology Palaeoecology; Suan et al., 2015, *Paleoceanography*; Erba et al., 2022, *Newsletters on Stratigraphy*), whereas most records display relatively consistent first-order T-OAE N-CIE trends that are robustly captured by our stacked LOESS smoothed record.

In addition, we have further quantified the magnitude of the N-CIE for each section and added the results to Supplementary Fig. 1B and Supplementary Table 1. Statistical analysis shows that the median N-CIE magnitude derived from individual sections (3.2‰; Supplementary Fig. 1B) is highly comparable to that obtained from the compiled and fitted global $\delta^{13}C_{carb}$ stack (3‰; Fig. 2B). Moreover, the violin plot demonstrates that the principal mode of the distribution is concentrated around ~3‰ (Supplementary Fig. 1B), indicating that most sections cluster within this range despite local variability.

Taken together, we consider that the compilation of 38 independent $\delta^{13}C_{carb}$ records provides a robust first-order representation of the global carbon-cycle perturbation during the T-OAE while reducing sensitivity to site-specific artefacts and outliers. Similar multi-record compilation approaches have also been widely used in studies of other hyperthermal events marked by major carbon-cycle perturbations to suppress regional noise and better resolve large-scale Earth system signals (e.g., Trampush and Hajek, 2017, *Geology*; Wu et al., 2021, *Nature Communications*). We have now clarified all these points in the revised manuscript.

-L 134 The calculation of d13C atm from the carbonate records should have some uncertainty that can be propagated forward.
We have now clarified this in the revised manuscript.

My secondary concern is the lack of agreement between model results and the stratigraphic record. Is this carbon flux (both emissions and burial) at all reasonable given what we know about the Karoo/Ferrar, magma/thermogenic volatile release, and carbon burial associated with the OAE?
We thank the reviewer for this insightful suggestion. We agree that, beyond comparisons among modelling studies, the plausibility of the reconstructed carbon release and burial should be evaluated against independent geological and stratigraphic constraints related to Karoo–Ferrar magmatism and the associated T-OAE carbon-cycle perturbation. At the same time, we note that geological constraints on carbon generation and release remain limited, particularly for the Ferrar province, where key parameters such as sill volumes, intrusion timescales, and host-rock distributions are still poorly constrained owing to the limited exposure of much of the province beneath the Antarctic ice sheet. We therefore do not regard any single geological estimate as definitive. Instead, we compare our model results with multiple independent geological, petrological, and stratigraphic lines of evidence to assess whether the reconstructed carbon-release masses and fluxes fall within a geologically plausible range. To address the reviewer’s concern, we have

substantially expanded the revised Discussion by adding a new section entitled "Geological and stratigraphic evidence for Karoo–Ferrar carbon release".

Specifically, we now compare our cGENIE-derived carbon-release estimates with several independent geological constraints. First, we emphasize the evidence for volcanism-related carbon release inferred from Hg records (e.g., Fendley et al., 2024, *Nature Geoscience*), which are derived from sedimentary Hg accumulation and volcanogenic Hg/CO_2 scaling relationships rather than Earth-system model inversions. We now explicitly compare these proxy-based estimates with our cGENIE-based carbon-release masses and fluxes.

Second, we have discussed some limited first-order petrological constraints on magmatic carbon inventories. Based on published estimates of Karoo–Ferrar magma volumes together with petrologically constrained magmatic CO_2 concentrations, we calculate a potential magmatic carbon inventory of approximately 7,400–23,900 Pg C. Although this represents the total carbon contained within the magma rather than the fraction ultimately degassed to the atmosphere, it provides an independent geological constraint on the plausible magnitude of magmatic carbon release.

Third, we have expanded the discussion of independent geological constraints on Karoo–Ferrar carbon release. In particular, we now incorporate published estimates of thermogenic carbon generation associated with sill emplacement and contact metamorphism. Basin-scale reconstructions based on intrusion geometry, host-rock distribution, and devolatilization processes suggest thermogenic carbon generation of ~7,500 Pg C. Although somewhat lower than our reconstructed cumulative carbon release, this estimate is broadly comparable in magnitude and supports the interpretation that intrusion-driven devolatilization constituted an important component of Karoo–Ferrar carbon release.

Meanwhile, we also discuss additional geological observations from both the Karoo and Ferrar provinces, including extensive intrusive systems and evidence for repeated magmatic activity, which together with published thermal-modelling studies indicate that volatile release likely occurred through multiple intrusive and degassing episodes rather than as a single event. These observations provide independent geological support for the temporally variable (multi-pulse) carbon-release pattern reconstructed by our inversion-based modelling. Because these geological observations primarily provide qualitative support for the style and timing of carbon release rather than additional quantitative constraints on carbon-release masses or fluxes, we sought to avoid distracting from the main focus of the manuscript and further increasing its length. We therefore retain only a brief summary in the main text and move the detailed discussion of Karoo–Ferrar intrusive systems, contact metamorphism, and associated geological evidence to a new Supplementary Text 3. This allows the main text to remain focused on the quantitative constraints provided by Hg-based reconstructions, magma-carbon inventories, thermogenic carbon

generation estimates, and Earth-system modelling, while preserving the full geological context in the Supplementary Information.

Finally, we have clarified the discussion of organic carbon burial and its relationship to the modelled carbon budget (please see the response below). Collectively, these geological, petrological, and stratigraphic constraints suggest that the reconstructed carbon-release masses and fluxes are compatible with the range permitted by currently available evidence, while also highlighting the need for improved geological constraints on the Karoo–Ferrar LIPs, particularly the Ferrar province, to further refine estimates of carbon generation and release during the T-OAE.

L330: -Is it then necessary to invoke an increase in OC burial earlier in the CIE (and hence larger C emissions to balance the effects of this burial)? I suspect that this could be prescribed, or at least the effects of this calculated (again this should be incorporated, at least descriptively, into the uncertainty reported for the model results – including in the figures. Right now, the carbon emissions/output seems invalidated by this observation. It is not sufficient, in my opinion, to say that the modelled flux is a "lower bound". Would this increase the proportion (or decrease the isotopic composition) of the necessary emissions? I think that a rigorous discussion of this is required in order for the model results to be useful – and indeed to demonstrate that majority magmatic carbon is sufficient (as claimed in the title).
We thank the reviewer for raising this important concern. We agree that enhanced OC burial may have begun before the interval of negative carbon flux reconstructed by the inversion model, and that additional burial during the onset of the N-CIE would require correspondingly larger carbon release to reproduce the observed N-CIE. Consequently, the reconstructed carbon-release masses, rates, and carbon-source compositions should be regarded as conservative estimates. At present, however, it is not possible to quantitatively incorporate such burial into the model framework. Although local TOC records provide evidence for changes in organic matter accumulation, they cannot readily be translated into a globally integrated OC burial flux suitable for prescribing in the model. We therefore regard the current inversion approach as the most robust first-order estimate presently achievable.

To evaluate whether omission of onset-phase burial could fundamentally alter our conclusions, **we now compile TOC records from 66 globally distributed sections and examine their temporal evolution across the event (Supplementary Fig. 6).** Although TOC begins to increase near the onset of the N-CIE, maximum enrichment is not reached until ~180 kyr after the initiation of the excursion, coincident with the recovery phase. This pattern suggests that OC burial intensified progressively through the event and may have been greatest during the interval already captured by the model. Furthermore, previous TOC-based estimates integrating the entire T-OAE suggest total additional OC burial of ~9,000 Pg C, only moderately larger than our modelled burial during the recovery interval alone. Together, these observations indicate that omission of burial during the onset phase likely leads to some

underestimation of carbon release, but probably not by an amount sufficient to fundamentally alter the principal conclusions of the study. We have clarified all these points in the revised manuscript.

-The negative CIE associated with the T-OAE is superimposed on a broader positive excursion (or at least shift – see e.g., Storm et al., 2020) – I find this hard to reconcile with the model results, in particular that increased organic burial is only invoked starting ~150 kyr after the n-CIE onset. Even if it is beyond scope to model this longer time interval, the timing of increased organic burial should be explicitly discussed (as noted in the previous comment).
Please see the response above.

-The Karoo/Ferrar LIP and thermogenic carbon release are discussed (e.g. L190), but these discussions are fairly abstract. Is it possible to use any of what we know about the volcanism or thermogenic volatile release to constrain or evaluate the model results in a quantitative way?
Please see the response above.

References

1. Beerling, D. J. & Brentnall, S. J. Numerical evaluation of mechanisms driving Early Jurassic changes in global carbon cycling. *Geology* **35,** 247–250 (2007).
2. Erba, E. et al. Carbon- and oxygen-isotope signature of the Toarcian Oceanic Anoxic Event: insights from two Tethyan pelagic sequences (Gajum and Sogno Cores–Lombardy Basin, northern Italy). *Newsl. Stratigr.* **55,** 451–477 (2022).
3. Fendley, I. M. et al. Early Jurassic large igneous province carbon emissions constrained by sedimentary mercury. *Nat. Geosci.* **17,** 241–248 (2024).
4. Heimdal, T. H., Goddéris, Y., Jones, M. T. & Svensen, H. H. Assessing the importance of thermogenic degassing from the Karoo Large Igneous Province (LIP) in driving Toarcian carbon cycle perturbations. *Nat. Commun.* **12,** 6221 (2021).
5. Kemp, D. B. et al. The timing and duration of large-scale carbon release in the Early Jurassic. *Geology* **52,** 891–895 (2024).
6. McElwain, J. C., Wade-Murphy, J. S. & Hesselbo, S. P. Changes in carbon dioxide during an oceanic anoxic event linked to intrusion into Gondwana coals. *Nature* **435,** 479–482 (2005).
7. Nordt, L., Breecker, D. & White, J. Jurassic greenhouse ice-sheet fluctuations sensitive to atmospheric CO_2 dynamics. *Nat. Geosci.* **15,** 54–59 (2022).
8. Röhl, H. J., Schmid-Röhl, A., Oschmann, W., Frimmel, A. & Schwark, L. The Posidonia Shale (Lower Toarcian) of SW-Germany: an oxygen-depleted ecosystem controlled by sea level and palaeoclimate. *Palaeogeogr. Palaeoclimatol. Palaeoecol.* **165,** 27–52 (2001).
9. Suan, G., Van De Schootbrugge, B., Adatte, T., Fiebig, J. & Oschmann, W. Calibrating the magnitude of the Toarcian carbon cycle perturbation. *Paleoceanography* **30,** 495–509 (2015).

10. Trampush, S. M. & Hajek, E. A. Preserving proxy records in dynamic landscapes: Modelling and examples from the Paleocene-Eocene Thermal Maximum. *Geology* **45,** 967–970 (2017).
11. Ullmann, C. V. et al. Warm afterglow from the Toarcian Oceanic Anoxic Event drives the success of deep-adapted brachiopods. *Sci. Rep.* **10,** 6549 (2020).
12. Wu, Y. et al. Six-fold increase of atmospheric pCO_2 during the Permian–Triassic mass extinction. *Nat. Commun.* **12,** 2137 (2021).

Reviewer #2 (Remarks to the Author):
Review of "massive release of predominantly magmatic carbon drove an Early Jurassic hyperthermal" by Chen et al., submitted to Nature Communications

I enjoyed reading the manuscript by Chen and co-authors. The manuscript is well written, easy to follow, and the authors clearly communicate the methods used, and the motivation behind the study. The study is robust and novel and provides constraints on carbon cycle dynamics during the T-OAE using the cGENIE model. Importantly, the authors take uncertainties of proxy records and carbon release (e.g., timing, isotopic signature, C mass/flux etc.) into account by providing sensitivity analyses, which is key in modeling studies. They also discuss simplifications, potential shortcomings and limitations in their study. Efforts in improving our understanding of past events of climatic and environmental disruptions, and the impacts from carbon sources, are important. The last section (implications for modern global warming) nicely ties their results, and the importance of understanding past climate/biotic disruptions, to the challenges we are facing in the present. I would be happy to recommend this manuscript for publication with minor changes. See comments below:
Line 88: Please specify that references for the isotope records can be found in Supplementary Table 1. Please also add this in the legend of Figure 1 and 2.
Revised as suggested.

Line 272: Could you please also add the experiment ID so that it is easy to compare to Supplementary Table 2. This comment applies not only here, but throughout the manuscript.
Revised as suggested.

Line 875: I recommend to separate panel A-B from C-F; I find it a bit confusing to show both model inputs and outputs in the same figure. I believe there are constraints on the number of figures allowed. Since Fig. 2 already shows model inputs; you could add the d13C curve from Fig. 3A to Fig. 2B, and the pCO2 curve in Fig. 3B to Fig 2A. Give the curves + uncertainty range a different color, dashed line etc. In addition to separating inputs from outputs + reducing the number of figure panels, this would also provide direct comparisons between the proxy data and the smoothed curves that you feed the model with.
We thank the reviewer for this constructive suggestion. Following this advice, we have now reorganized Figures 2 and 3 to improve clarity by separating model forcings (inputs) from model results (outputs).

Line 886: It is a bit difficult to see the temperature increases due to the large range in values. Removing all white space would be helpful – for example you can start your y-axis at 10 degrees C.
Revised as suggested.

Line 320: I would recommend not to abbreviate the end-Permian mass extinction.
Revised as suggested.

Reviewer #2 (Remarks on code availability):

When I click the link I am not given access, so I have not been able to review the code.
We apologize for the issue with code availability. We have now re-uploaded the code and all files related to the simulations.

Response letter for NCOMMS-26-016106B: Massive release of predominantly magmatic carbon drove an Early Jurassic hyperthermal

We thank the editor and the reviewers for their constructive comments on our manuscript. Following their suggestions, we have carefully addressed the two queries raised (shown in black text) and provide detailed, point-by-point responses below (in blue text).

Reviewer #1 (Remarks to the Author):

The authors have thoughtfully responded to my critique and have made a significant effort to address it, including the addition of the TOC compilation, additional uncertainty estimates and propagation, and comparison with other published models and TOAE carbon emissions.

I have a few outstanding comments.

"These results help to underline that uncertainties associated with reconstructed pCO2 values during the T-OAE exert only a limited influence on modelled carbon-release histories and source characteristics"

This makes it seem like the required model forcing you need is not very sensitive to the pco2 constraint. Is that the case?

We thank the reviewer for raising this important point. We agree that our previous wording understated the sensitivity of the reconstructed carbon-release magnitude to the pCO_2 used in the modelling.

Relative to the standard experiment (which uses the smoothed median pCO_2 data, experiment 2), the maximum-pCO_2 scenario (experiment 4) increases cumulative carbon release from ~18,000 to 26,000 Pg C. The peak carbon-release rate also increases from ~0.16 to 0.21 Pg C yr^{-1}. The flux-weighted mean $\delta^{13}C_{source}$ shifts from −13.6‰ in experiment 2 to −10.7‰ in experiment 4. These results demonstrate that the inferred mass and rate of carbon release are sensitive to the magnitude of the imposed pCO_2 forcing. Note that across all pCO_2 scenarios (experiments 1–4), our modelled masses and rates of carbon release (Supplementary Table 2) fall within the much wider range obtained from previous modelling work.

Importantly, despite the greater cumulative release and peak rate, experiment 4 preserves the multi-pulse structure of carbon input. Moreover, the higher flux-weighted mean $\delta^{13}C_{source}$ obtained in experiment 4 is still consistent with a dominant contribution from relatively ^{13}C-enriched magmatic carbon, and hence does not alter our conclusions.

We have revised the relevant discussion in the main text and Supplementary Information accordingly, and have removed wording implying that the influence of pCO_2 uncertainty is 'limited'.

L405: Consequently, although OC burial during the N-CIE onset phase is not captured by our inversion approach, available stratigraphic records suggest that it likely constituted a smaller component of total event-scale OC burial than that

associated with the recovery interval, and therefore is unlikely to substantially affect our model results.

Is it possible to do some kind of quantitative analysis, so it can be better than “unlikely”? How affected might it be?

We thank the reviewer for this important suggestion. We agree that our previous qualitative statement that onset OC burial was “unlikely” to substantially affect the model did not adequately quantify the potential bias introduced by the inversion framework.

A fully independent reconstruction of global onset OC burial from the available stratigraphic records is not presently possible. Such a reconstruction would require spatially resolved depositional areas, knowledge of dry bulk-rock densities, sedimentation rates, organic matter preservation efficiencies and information on hiatuses, which are unavailable for most sections. Nevertheless, we agree that these uncertainties should not preclude a first-order quantitative assessment, which we have now incorporated into our revised manuscript.

In brief, we have now performed a stratigraphically informed, N-CIE thickness-weighted sensitivity analysis to estimate the temporal partitioning of net excess OC burial between the T-OAE N-CIE onset and recovery intervals. Full details of this sensitivity analysis are provided in our revised Methods.

The results of our analysis suggest that ~30% of the total OC burial during the T-OAE could have occurred during the onset interval, with the remaining ~70% occurring during the recovery interval. Applying this partitioning to the whole-event estimate of ~9,000 Pg C of net additional OC burial reported by Kemp et al. (2022) suggests that ~2,700 Pg C was buried in the onset, and ~6,300 Pg C was buried in the recovery. This latter estimate is close to our estimate of ~6,800 Pg C buried during the recovery interval in experiment 2.

If the omitted onset OC burial were offset by an equal amount of additional carbon release, the cumulative release required by the standard experiment would increase from approximately 18,000 to 20,500 Pg C. Distributed uniformly over the 100 kyr onset interval, the omitted sink is equivalent to a mean compensating flux of ~0.03 Pg C yr^{-1}. This interval-average sensitivity is not added directly to the modelled peak emission rate because the detailed temporal distribution of onset burial is not independently constrained.

We also evaluated the first-order isotope-mass-balance consequence of the omitted onset sink. The mean isotopic composition of buried OC during the T-OAE is not constrained, but Phanerozoic black shales have a mean $\delta^{13}C_{org}$ of −27‰ (Meyers, 2014). We therefore use −27‰ as a representative value. Under the assumption that an additional carbon input would exactly compensate both the mass and isotopic effect of the omitted burial (i.e., mass balance), the whole-event flux-weighted $\delta^{13}C_{source}$ shifts from −13.6‰ to −15.4‰.

These calculations indicate that omission of onset OC burial leads to an

underestimate of cumulative carbon release. Accounting for this sink increases the estimated gross release by ~15% and requires a moderately lighter whole-event source composition, but it does not affect our conclusion that the T-OAE carbon-cycle perturbation required a dominant contribution from relatively ^{13}C-enriched magmatic carbon.

All the above results are now described in our revised manuscript. We also emphasize in our revision that this calculation is only a conditional sensitivity analysis rather than a new global OC burial reconstruction or a rerun of the cGENIE inversion. The absolute Pg C estimates remain conditional on the published 9,000 Pg C whole-event burial estimate, which is approximate. The quantitative analysis has been added to the "Modelled carbon sequestration during the T-OAE" section and the Methods. We do not incorporate the corrected values into Fig. 3 or the formal cGENIE uncertainty envelope because this would conflate direct inversion outputs with an external scaling conditional on the published 9,000 Pg C estimate.

References

1. Kemp, D. B., Suan, G., Fantasia, A., Jin, S. & Chen, W. Global organic carbon burial during the Toarcian oceanic anoxic event: Patterns and controls. *Earth-Sci. Rev.* **231,** 104086 (2022).
2. Meyers, P. A. Why are the $\delta^{13}C_{org}$ values in Phanerozoic black shales more negative than in modern marine organic matter? *Geochem. Geophys. Geosyst.* **15,** 3085–3106 (2014).
